# Electrolyte-anion-controlled reactivity of aromatic radical cations

**DOI:** 10.1039/d6sc00891g

**Published:** 2026-05-19

**Authors:** Naoki Shida, Takuma Maekawa, Yuki Yasuno, Su-Gi Chong, Ikuyoshi Tomita, Mahito Atobe, Shinsuke Inagi

**Affiliations:** a Department of Chemistry and Life Science, Yokohama National University 79-5 Tokiwadai, Hodogaya-ku Yokohama 240-8501 Japan shida-naoki-gz@ynu.ac.jp atobe-mahito-wk@ynu.ac.jp; b Institute of Advanced Sciences, Yokohama National University 79-5 Tokiwadai, Hodogaya-ku Yokohama 240-8501 Japan; c PRESTO, Japan Science and Technology Agency (JST) 4-1-8 Honcho, Kawaguchi Saitama 332-0012 Japan; d Department of Chemical Science and Engineering, School of Materials and Chemical Technology, Institute of Science Tokyo Nagatsuta-cho 4259, Midori-ku Yokohama 226-8501 Japan inagi@mct.isct.ac.jp

## Abstract

Electrolytes are indispensable in electrosynthesis, yet their function is generally confined to ionic conduction. Here we show that specific association of electrolyte anions provides a powerful means to regulate the reactivity of electrogenerated aromatic radical cations. Electrochemical oxidation of 2,5-diarylthiophenes in the presence of a coordinating anion, triflate (TfO^−^), selectively triggers C–C bond formation, leading to dimerization followed by oxidative cyclization to afford thiophenium cations. In contrast, weakly coordinating anions such as B(C_6_F_5_)_4_^−^ stabilize persistent and largely unreactive radical cations. Spectroscopic analyses confirm radical cation formation under weakly coordinating conditions, while the presence of a coordinating anion fundamentally alters the reaction pathway, selectively enabling productive C–C bond formation. These results establish electrolyte anion coordination as a general strategy to switch between reaction pathways, offering a new design principle for selective electrosynthetic transformations.

## Abstract 2

Electrolyte anions actively control the fate of aromatic radical cations, switching them between persistence and productive C–C bond formation.

## Graphical abstract

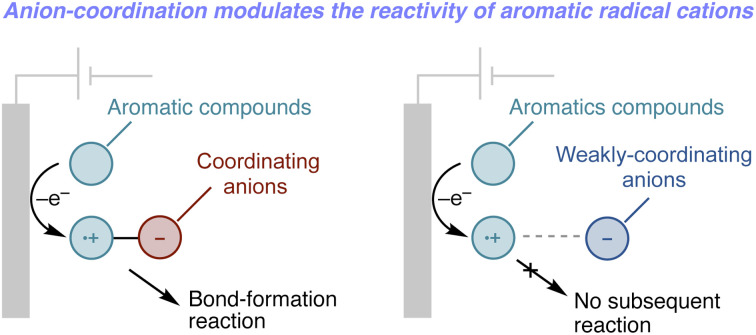

## Introduction

**Fig. 1:**
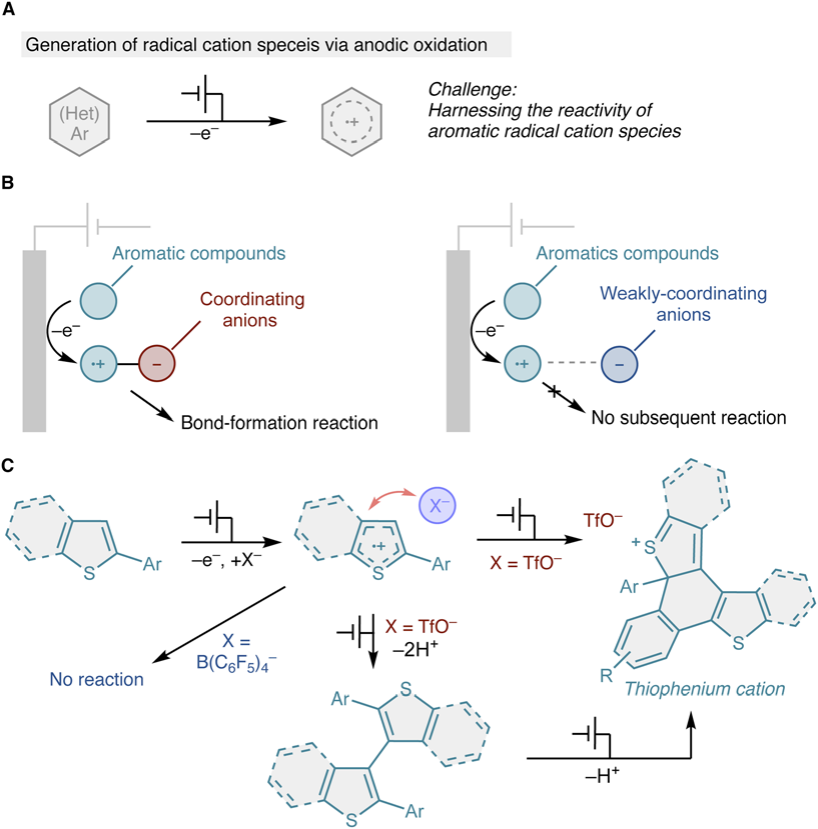
Conceptual overview of this work. (A) Reactivity control of aromatic radical cations. (B) Electrolyte-anion-controlled switching of radical cation reactivity. (C) Oxidative C–C bond formation and cyclization of diarylthiophene derivatives.

Electrochemical oxidation offers a sustainable and tunable platform for organic synthesis by enabling access to high-energy intermediates without the use of stoichiometric oxidants.^1–3^ Among these intermediates, aromatic radical cations generated *via* single-electron transfer are particularly attractive because of their pronounced electrophilicity and unique reaction pathways.^4–11^ However, controlling the reactivity of π-delocalized radical cations remains a fundamental challenge: once formed, these open-shell species often undergo indiscriminate follow-up reactions or persist without productive bond formation, thereby limiting synthetic selectivity (Fig. 1A).

To date, strategies to regulate electrochemical reactivity have largely focused on electrode materials, electrical potential, and solvent effects.^12–14^ In contrast, supporting electrolytes are most often treated as passive components that merely ensure ionic conduction and charge balance. However, this conventional view has long been known to be incomplete. Classical studies in aromatic electrochemistry have shown that electrolyte identity and ion-pairing can influence redox potentials, electron-transfer kinetics, and product distributions.^15–17^ More recent discussions have further emphasized that supporting electrolytes can play roles beyond conductivity.^18–22^

Despite these important precedents, systematic experimental demonstrations of how electrolyte anions switch the fate of electrogenerated aromatic radical cations between persistence and productive bond formation remain limited. In particular, direct comparison of weakly coordinating and more strongly coordinating anions within a defined substrate class, combined with spectroscopic observation of radical-cation accumulation and preparative validation of downstream C–C bond formation, has not been fully established.

Building on these precedents, we asked whether electrolyte-anion effects could be translated into a more deliberate strategy for controlling the lifetime and follow-up reactivity of aromatic radical cations (Fig. 1B). Specifically, a coordinating anion may engage an electrogenerated aromatic radical cation through specific anion association, perturbing its electronic structure and lowering the kinetic barrier for productive bond-forming pathways. Conversely, weakly coordinating anions^23^ may stabilize the radical cation state, favoring its persistence over follow-up chemistry.

Herein, we demonstrate that electrolyte anion coordination decisively governs the reactivity of radical cations generated from π-extended thiophene derivatives. Using triflate (TfO^−^) as a coordinating anion, anodic oxidation selectively promotes C–C bond formation, leading to dimerization and subsequent oxidative cyclization to form thiophenium cations *via* an overall two-electron process (Fig. 1C). In contrast, weakly coordinating anions (B(C_6_F_5_)_4_^−^) stabilize persistent and largely unreactive radical cations. These findings establish electrolyte anion coordination as a general strategy to modulate radical cation reactivity and selectively access bond-forming pathways in organic electrosynthesis.

## Results and discussion

**Fig. 2:**
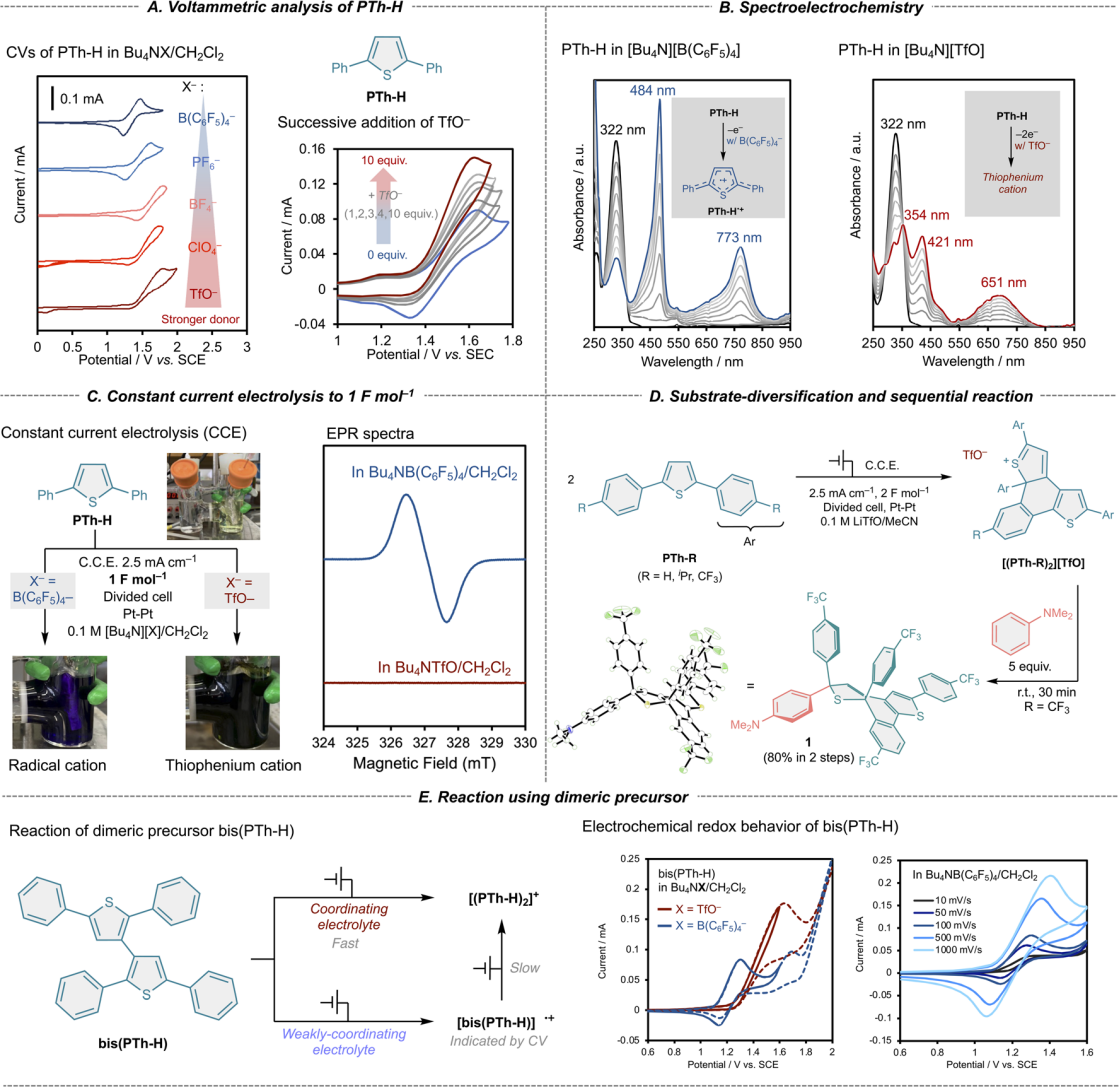
Electrochemical behaviour of PTh-H in the presence of coordinating and weakly coordinating anions. (A) Cyclic voltammograms of PTh-H in CH_2_Cl_2_ containing various supporting electrolytes. (B) UV-vis spectroelectrochemical spectra recorded under anodic oxidation. (C) Bulk electrolysis and EPR analysis under weakly coordinating and coordinating conditions. (D) Substrate diversification of PTh-R and subsequent transformation. (E) Electrochemical oxidation of independently synthesised bis(PTh-H).

Cyclic voltammetry (CV) of 2,5-diphenylthiophene (PTh-H) was first examined in dichloromethane (Gutman's donor number: CH_2_Cl_2_ = 1)^24^ using a series of tetrabutylammonium salts with counter anions of varying donor strength (Fig. 2A).^25^ When weakly coordinating anions such as B(C_6_F_5_)_4_^−^ and PF_6_^−^ were employed, PTh-H exhibited a fully reversible one-electron oxidation. In contrast, progressively less reversible behavior was observed with increasing anion donor number, and in the presence of the strongly coordinating triflate anion (TfO^−^), the oxidation became completely irreversible and was accompanied by a pronounced increase in anodic current. These results indicate that the electrochemical behavior of PTh-H is highly sensitive to the identity of the supporting electrolyte anion.

The influence of anion coordination was further confirmed by incremental addition of Bu_4_NTfO to a solution of PTh-H in a weakly coordinating electrolyte (Bu_4_NPF_6_/CH_2_Cl_2_). Even small amounts of TfO^−^ markedly transformed the voltammetric response from a reversible one-electron process to an irreversible oxidation with enhanced current (Fig. 2A, right), demonstrating that TfO^−^ directly perturbs the fate of the oxidized species rather than acting as a passive spectator.

To identify the electrochemically generated intermediates, spectroelectrochemical measurements were performed (Fig. 2B). In the presence of the weakly coordinating B(C_6_F_5_)_4_^−^ anion, oxidation of PTh-H produced characteristic absorption bands at 770 and 490 nm, which are assigned to the HOMO–SOMO and SOMO–LUMO transitions of the radical cation PTh-H˙^+^.^20^ By contrast, oxidation in the presence of TfO^−^ resulted in a distinct spectral profile with absorption maxima at 690 and 430 nm, indicating the formation of a different cationic species. These observations suggest that although initial electron transfer occurs under both conditions, the subsequent chemical fate of the oxidized intermediate is strongly dependent on anion coordination.

Bulk electrolysis experiments provided decisive insight into this divergence (Fig. 2C). Electrolysis of PTh-H under weakly coordinating conditions (Bu_4_NB(C_6_F_5_)_4_/CH_2_Cl_2_) with passage of 1 F mol^−1^ of charge yielded a purple solution whose UV-vis and EPR spectra were fully consistent with the persistent radical cation PTh-H˙^+^. In contrast, electrolysis under coordinating conditions (Bu_4_NTfO/CH_2_Cl_2_) afforded a green solution that was EPR silent despite the same charge being passed. ^1^H NMR analysis revealed the presence of both unreacted PTh-H and a new dimeric species, identified as the thiophenium salt [(PTh-H)_2_][TfO]. Notably, formation of this product formally requires a two-electron oxidation per PTh-H unit, indicating that successive oxidation is favored over accumulation of the radical cation in the presence of TfO^−^.

Consistent with this interpretation, electrolysis under coordinating conditions with passage of 2 F mol^−1^ of charge resulted in clean and quantitative formation of [(PTh-H)_2_][TfO] as the sole detectable species (Fig. 2D). The structure of the thiophenium salt was established by multinuclear NMR and mass spectroscopy (see SI). These results demonstrate that coordination of TfO^−^ modulates the intrinsic reactivity of the radical cation generated by one-electron oxidation of PTh-H, thereby enabling subsequent bond-forming reactions that lead to an overall two-electron transformation.

This anion-controlled transformation was also observed for a series of related substrates (Fig. 2D). Electrochemical oxidation of symmetrical biarylthiophenes bearing –^i^Pr or –CF_3_ substituents at the *para*-position of the aryl groups (PTh-^i^Pr, and PTh-CF_3_) under coordinating conditions led to clean formation of the corresponding thiophenium salts.

To unambiguously establish the structure of thiophenium salts, a subsequent chemical transformation was carried out using [(PTh-CF_3_)_2_][TfO] (Fig. 2D). Upon addition of *N*,*N*-dimethylaniline to the electrochemically generated [(PTh-CF_3_)_2_][TfO] without purification, the characteristic green coloration disappeared immediately. The product obtained in 80% yield was subjected to single-crystal X-ray diffraction analysis, which confirmed the formation of compound 1, a Pummerer-type reaction product. Since Pummerer reactions are characteristic transformations of thiophenium and thionium salts,^26–28^ this result provides compelling evidence that [(PTh-CF_3_)_2_][TfO] indeed possesses the expected thiophenium-type structure.

To probe whether anion coordination also influences the subsequent cyclization step, the independently synthesized dimeric precursor bis(PTh-H) was subjected to electrochemical oxidation (Fig. 2E). In the presence of TfO^−^, bis(PTh-H) underwent irreversible oxidation to afford the corresponding thiophenium cation, consistent with a coordination-assisted two-electron process. In contrast, oxidation in the presence of the weakly coordinating B(C_6_F_5_)_4_^−^ anion exhibited partially reversible behavior, with scan-rate-dependent irreversibility becoming more pronounced at slower scan rates (Fig. 2E). These observations suggest that anion coordination not only governs the initial oxidation state of PTh-H but also facilitates the oxidative cyclization of the dimeric intermediate.

Taken together, the combined electrochemical, spectroscopic, and bulk electrolysis data establish that specific association of TfO^−^ fundamentally alters the reaction pathway of electrogenerated PTh-H˙^+^, selectively promoting C–C bond formation *via* an overall two-electron oxidation process.

**Fig. 3:**
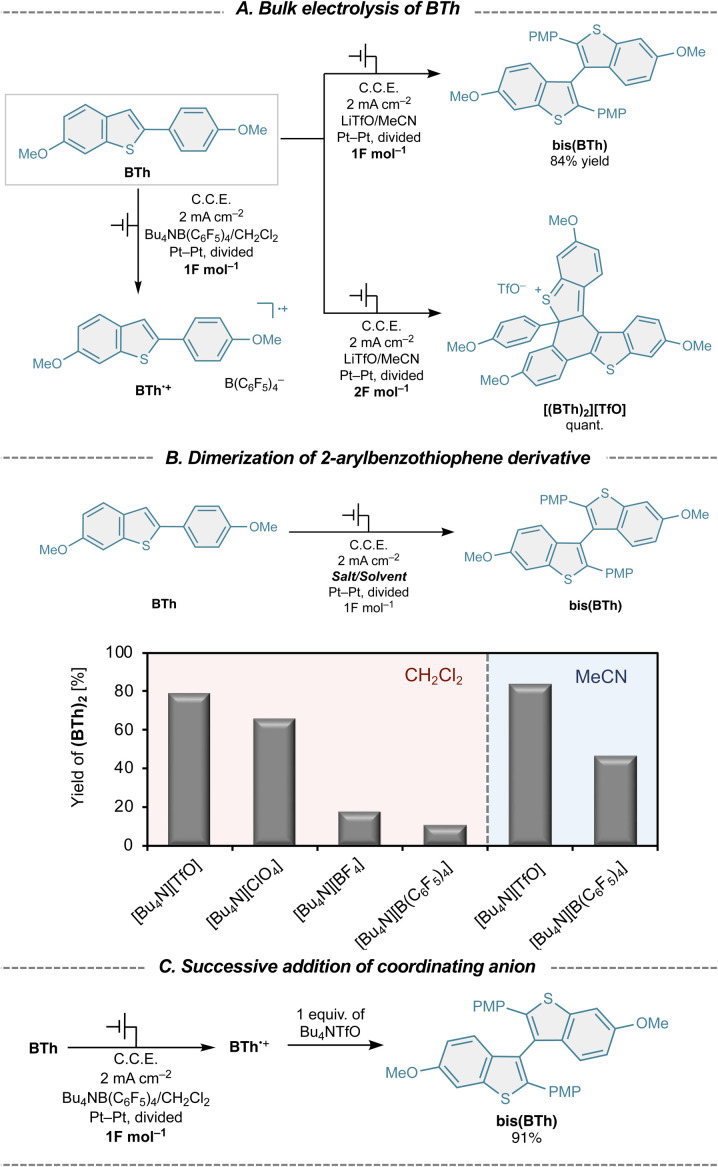
Electrochemical oxidation of BTh under coordinating and weakly coordinating conditions. (A) Bulk electrolysis of BTh in CH_2_Cl_2_ using different supporting electrolytes. (B) Yield of bis(BTh) as a function of the electrolyte anion. (C) Electrochemical generation of BTh˙^+^ under weakly coordinating conditions, followed by post-electrolysis addition of a coordinating anion.

To further elucidate the role of anion coordination in the intermolecular C–C bond-forming step, we next investigated the electrochemical oxidation of benzo[*b*]thiophene derivative, BTh, as a mechanistically simpler model system (Fig. 3). Unlike PTh-H, BTh undergoes selective intermolecular dimerization prior to cyclization, allowing direct interrogation of the C–C coupling process. Under weakly coordinating conditions, anodic oxidation of BTh generated the corresponding radical cation BTh˙^+^, as confirmed by spectroscopic analysis, but no productive bond formation was observed. In contrast, electrolysis in the presence of the coordinating TfO^−^ anion resulted in efficient formation of the 3,3′-dimer bis(BTh) upon passage of 1 F mol^−1^ of charge (Fig. 3A). When 2 F mol^−1^ of charge was passed under otherwise identical conditions, the corresponding thiophenium salt [(BTh)_2_][TfO] was obtained selectively. These results indicate that, for BTh, intermolecular dimerization precedes oxidative cyclization, in contrast to the partially overlapping processes observed for PTh-H.

The effect of electrolyte anion identity on the dimerization step was systematically examined (Fig. 3B). Electrolysis of BTh (1 F mol^−1^) in dichloromethane revealed a strong dependence of dimer yield on anion donor strength, with TfO^−^ providing the highest efficiency. In sharp contrast, the weakly coordinating B(C_6_F_5_)_4_^−^ anion predominantly stabilized the radical cation, suppressing C–C bond formation. These observations indicate that anion coordination is a critical factor in enabling productive intermolecular coupling of BTh radical cations. Solvent effects further support this conclusion. When acetonitrile, a coordinating solvent (Gutman's donor number: MeCN = 14.1),^24^ was employed, dimerization occurred even in the presence of B(C_6_F_5_)_4_^−^, although the yield remained significantly higher with TfO^−^. This behavior suggests that coordination plays a decisive role in promoting C–C bond formation from radical cation intermediates.

To directly probe whether TfO^−^ interacts with the radical cation during the coupling step, a cation-pool approach was employed.^29^BTh˙^+^ was first generated electrochemically under weakly coordinating conditions by passage of 1 F mol^−1^ of charge, after which electrolysis was halted and Bu_4_NTfO was added (Fig. 3C). Remarkably, this post-electrolysis addition of TfO^−^ resulted in rapid and near-quantitative formation of bis(BTh) (91% yield). This experiment clearly demonstrates that TfO^−^ engages with BTh˙^+^ after electron transfer, actively facilitating intermolecular C–C bond formation rather than merely influencing the initial oxidation event.

Importantly, the quantitative formation of bis(BTh) upon passage of exactly 1 F mol^−1^ of charge establishes that dimerization proceeds through an EC mechanism involving coupling of two radical cations, rather than an ECEC pathway. Taken together, the BTh system provides compelling evidence that coordinating anions act catalytically to unlock otherwise inaccessible radical cation coupling pathways, reinforcing the generality of anion-controlled switching of radical cation intermediates.

**Fig. 4:**
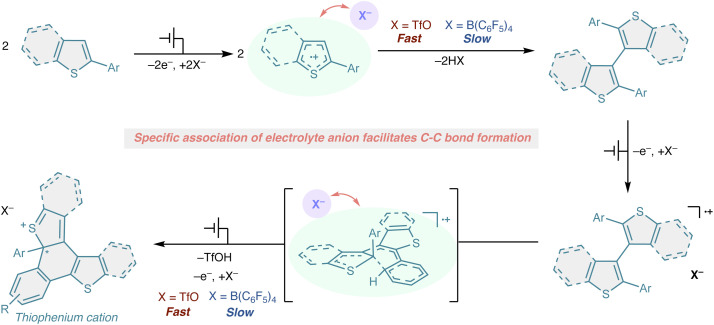
Plausible reaction mechanism.

On the basis of the experimental observations described above, we propose a mechanism in which coordination of a supporting electrolyte anion modulates the intrinsic reactivity of electrogenerated radical cations (Fig. 4). Under weakly coordinating conditions, anodic oxidation of PTh-R proceeds *via* a clean one-electron process to generate the corresponding radical cation. Owing to the π-extended framework of the diarylthiophene scaffold, this radical cation is sufficiently stabilized to persist without engaging in productive follow-up reactions. In the presence of a coordinating anion such as TfO^−^, the initial electron-transfer step similarly generates a radical cation. However, specific association of TfO^−^ to this open-shell intermediate perturbs its electronic structure, destabilizing the nonreactive radical cation state and rendering it susceptible to C–C bond-forming reactions. As a consequence, radical cation–radical cation coupling becomes kinetically accessible, leading to intermolecular C–C bond formation. Importantly, the effect of TfO^−^ coordination is not limited to intermolecular coupling. The same anion-induced activation also accelerates the subsequent intramolecular oxidative cyclization of the dimeric intermediate. In this context, TfO^−^ does not act as a stoichiometric reagent but rather as a promoter that facilitates both inter- and intramolecular C–C bond formation from radical cation intermediates. It should be noted that the role of TfO^−^ in this process may not be limited to modulation of the radical-cation state through coordination. Because C–C bond formation is accompanied by deprotonation, TfO^−^ may also assist proton transfer as a local proton acceptor after association with the cationic radical manifold. Protons released during C–C bond formation and oxidative cyclization are expected to be transferred as proton equivalents through the non-aqueous electrolyte and eventually reduced at the cathode, most likely to form H_2_.

Taken together, these findings suggest that coordinating anions such as TfO^−^ function as catalysts in radical cation chemistry, modulating the reactivity landscape of open-shell intermediates and enabling bond-forming pathways that are otherwise disfavored under electrochemical conditions.

## Conclusions

In conclusion, we have demonstrated that coordination of supporting electrolyte anions provides a powerful means to regulate the reactivity of electrogenerated radical cations. Using 2,5-diarylthiophenes as a model system, we showed that weakly coordinating anions stabilize persistent radical cations, whereas specific association of triflate (TfO^−^) electronically activates these open-shell intermediates, enabling both intermolecular C–C coupling and subsequent intramolecular oxidative cyclization.

Importantly, the role of the coordinating anion is not to directly induce a second electron-transfer event but to modulate the intrinsic reactivity of radical cations, rendering otherwise disfavored bond-forming pathways kinetically accessible under electrochemical conditions. In this context, the electrolyte anion functions in a catalytic manner, controlling the reaction landscape without being consumed.

More broadly, this work establishes electrolyte design as a general and conceptually simple approach to controlling radical cation reactivity in organic electrosynthesis. We anticipate that this principle will be broadly applicable to other classes of organic redox transformations, providing new opportunities for selective bond formation under electrochemical conditions.

## Author contributions

N. S., M. A., and S. I. coordinated the project. N. S., M. A., and S. I. conceived the project, designed the experiments, and wrote the manuscript. T. M. and Y. Y. carried out the synthetic experiments. S.-G. C. supported analysis and characterization. N. S., I. T., M. A., and S. I. contributed to discussing of the reaction mechanism and analysis of synthetic results.

## Conflicts of interest

There are no conflicts to declare.

## Supplementary Material

SC-OLF-D6SC00891G-s001

SC-OLF-D6SC00891G-s002

## Data Availability

The data presented in this study are available upon reasonable request from the corresponding authors. CCDC 2526613 contains the supplementary crystallographic data for this paper.^30^ Supplementary information: all experimental procedures and characterisation data of all synthesized compounds. See DOI: https://doi.org/10.1039/d6sc00891g.
