# Supplementary material for: Electrolyte-anion-controlled reactivity of aromatic radical cations
Source: Chem Sci. 2026 May 19;17(25):12307–12. doi: 10.1039/d6sc00891g (PMC13185756; doi:10.1039/d6sc00891g)
Supplement: SC-017-D6SC00891G-s001 [file SC-017-D6SC00891G-s001.pdf]

Supplementary Information (SI) for Chemical Science.
This journal is © The Royal Society of Chemistry 2026

Supporting Information

Electrolyte-Anion-Controlled Reactivity of Aromatic Radical Cations

Naoki Shida,[1,2,3] Takuma Maekawa,[4] Yuki Yasuno,[1] Su-Gi Chong,[2] Ikuyoshi Tomita,[4] Mahito Atobe, [1,2,]* Shinsuke Inagi[4]**

[1] Department of Chemistry and Life Science, Yokohama National University, 79-5 Tokiwadai, Hodogaya-ku, Yokohama 240-8501, Japan

[2] Institute of Advanced Sciences, Yokohama National University, 79-5 Tokiwadai, Hodogaya-ku, Yokohama 240-8501, Japan.

[3] PRESTO, Japan Science and Technology Agency (JST), 4-1-8 Honcho, Kawaguchi, Saitama 332-0012, Japan

[4] Department of Chemical Science and Engineering, School of Materials and Chemical Technology, Institute of Science Tokyo, Nagatsuta-cho 4259, Midori-ku, Yokohama 226-8501, Japan

Table of Content

1. *General considerations* *S2*
2. *Synthesis* *S3*
3. *Summary of reported donor number data for solvents and anions* *S5*
4. *Detailed experimental description of Figure 2* *S6*
5. *Detailed experimental description of Figure 3* *S12*
6. *Single crystal X-ray diffraction* *S15*
7. *Supporting reference* *S17*
8. *NMR charts* *S18*

1. General considerations

Reagents and dry solvents were obtained commercially and used without further purification. Reactions were performed under an inert atmosphere with the Schlenk technique unless otherwise noted. ^{1}H, ^{13}C and ^{19}F NMR spectra were recorded on JEOL ECA400 (^{1}H: 399.78 MHz, ^{13}C: 100.53 MHz, ^{19}F: 376.46MHz), JEOL ECA500 (^{1}H: 500 MHz, ^{13}C: 126 MHz) and JEOL AVANCE NEO 500 (^{13}C: 126 MHz) spectrometers using $CDCl_3$ or CD_3CN as a solvent. The chemical shifts for ^{1}H, ^{13}C, and ^{19}F NMR spectra are given in δ (ppm) relative to internal tetramethylsilane, deuterated solvent, and monofluorobenzene (^{19}F NMR: –113.50 ppm), respectively. Multiplicities are abbreviated as singlet (s), doublet (d), doublet of doublets (dd), multiplet (m), and broad (br). Electrospray-ionization time-of-flight mass spectra (ESI-TOF MS) were obtained on a Bruker Daltonics micrOTOF focus II. Electron paramagnetic resonance (EPR) spectroscopy measurements were conducted using Electron Spin Resonance Spectrometer JES-FA200 X-band (JEOL). The single crystal X-ray analyses were carried out on a Rigaku XtaLAB Synergy-DW (with) Hybrid Photon Counting (HPC) detector (Cu Kα radiation, λ = 1.54184 Å). An empirical absorption correction was carried out by the MULTI-SCAN method. The structures were solved employing a dual-space algorithm, SHELXT (SHELX2014), and refined through a full-matrix least-squares method (SHELXL).[2,3] The non-hydrogen atoms were refined anisotropically. Hydrogen atoms were purified using the riding model. UV-vis absorption spectra were recorded on a SHIMAZU UV-1800. Cyclic voltammetry (CV) measurements were performed using ALS Instruments model 2325 or VSP-3A (Biologic). All CV measurements were carried out in the three-electrode system equipped with a platinum (Pt) disk working electrode, a Pt plate counter electrode (10 mm × 10 mm), and a saturated calomel electrode (SCE) or $Ag/AgNO_3$ reference electrode, which were calibrated to SCE value according to the ferrocene/ferrocenium redox couple.

2. Synthesis

*2-1. Synthesis of **PTh-CF₃***

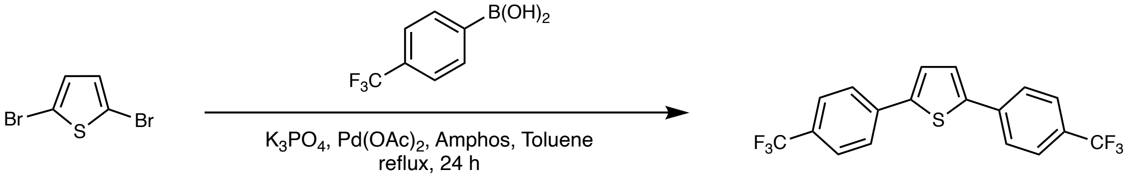

2,5-Dibromothiophene (441.3 mg, 1.7 mmol), 4-trifluoromethylphenylboronic acid (703 mg, 3.7 mmol), K_3PO_4 (2.9 g, 13.6 mmol), $Pd(OAc)_2$ (19 mg, 0.085 mmol), Amphos (45 mg, 0.17 mmol) were dissolved in toluene (10 mL) under a nitrogen atmosphere and the mixture was refluxed for 24 h. After cooling to room temperature, the reaction was quenched by adding deionized water, and the resulting mixture was extracted with dichloromethane. The organic layer was washed with water and dried over $MgSO_4$. After filtration and removal of the solvent, the residue was purified by silica gel column chromatography using hexane/dichloromethane (8/2) to give a yellow solid (324 mg, 58%). ^{1}H NMR spectrum corresponded to the reported data.[1]

*2-2. Synthesis of **PTh-iPr***

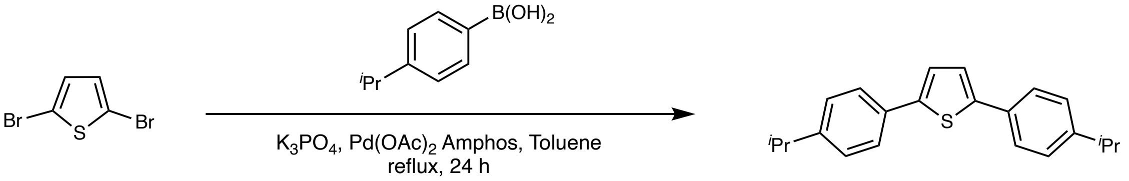

2,5-Dibromothiophene (1243 mg, 5.0 mmol), 4-isopropylphenylboronic acid (1968 mg, 12 mmol), K_3PO_4 (8491 mg, 40 mmol), $Pd(OAc)_2$ (56 mg, 0.25 mmol), Amphos (132.7 mg, 0.5 mmol) were dissolved in toluene (30 mL) under a nitrogen atmosphere and the mixture was refluxed for 24 h. After cooling to room temperature, the reaction was quenched by adding deionized water, and the resulting mixture was extracted with dichloromethane. The organic layer was washed with water and dried over $MgSO_4$. After filtration and removal of the solvent, the residue was purified by silica gel column chromatography using hexane/dichloromethane (8/2) to give a yellow solid (390 mg, 24%).

PTh-^{i}Pr: ^{1}H NMR (399.78 MHz, $CDCl_3$, ppm): δ = 7.55 (d, J = 8.2 Hz, 4H), 7.24 (d, J = 8.2 Hz, 2H, Ar), 2.92 (sep, J = 6.9 Hz, 2H), 1.27 (d, J = 6.9 Hz, 12H); ^{13}C NMR (126 MHz, $CDCl_3$, ppm) δ = 148.4, 143.3, 132.2, 127.1, 125.8, 123.6, 34.0, 24.1; HRMS (APCI-TOF): calcd for $C_{26}H_{33}S^+$ ($[M+H]^+$) m/z 321.17; found 321.1671.

*2-3. Synthesis of **bis(PTh-H)***

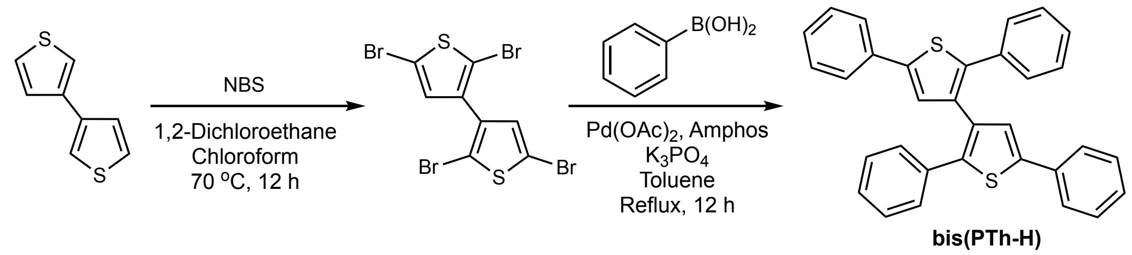

Bis(PTh-H) was obtained from 3,3'-bithiophene in two steps.

3,3'-Bithiophene (1.25g, 7.5 mmol) was reacted with *N*-bromosuccinimide (NBS, 8.0g, 45 mmol) in a mixed solvent of 1,2-dichloroethane (50 mL) and chloroform (50 mL) at 70 °C for 12 h under vigorous stirring. After cooling to room temperature, the reaction solution was evaporated under reduced pressure, redissolved in dichloromethane, and washed with deionized water and brine. The organic layer was dried over $MgSO_4$, filtered, and removed the solvent in vacuo. The desired product, 2,2',5,5'-tetrabromo-3,3'-bithiophene, was obtained as an orange solid (3.4g, 7.1 mmol, 95% yield).

2,2',5,5'-tetrabromo-3,3'-bithiophene: ^{1}H NMR (500 MHz, $CDCl_3$, ppm): 7.02 (s, 2H); ^{13}C NMR (126 MHz, $CDCl_3$, ppm): δ = 135.1, 131.4, 111.3, 111.0.

2,2',5,5'-Tetrabromo-3,3'-bithiophene (2.5 g, 5.2 mmol), phenylboronic acid (3.2 g, 26 mmol), K_3PO_4 (19.7 g, 92.8 mmol), $Pd(OAc)_2$ (58 mg , 0.26 mmol), Amphos (138 mg, 0.52 mmol) were dissolved in toluene (50 mL) under a nitrogen atmosphere and the mixture was refluxed for 12 h. After cooling to room temperature, the reaction solution was filtered, and the filtrate was evaporated under reduced pressure. The crude mixture was redissolved in chloroform and was washed with water. The organic layer was dried over $MgSO_4$. After filtration and removal of the solvent, the crude material was dissolved in $CHCl_3$ (20 mL) and was added dropwise into hexane (300 mL). The precipitate was filtered, and the residual solid was collected and dried in vacuo. The desired product, bis(PTh-H), was obtained as a pale green solid (0.94 g, 2 mmol, 38% yield).

Bis(PTh-H): ^{1}H NMR (500 MHz, $CDCl_3$, ppm): δ = 7.58–7.56 (m, 4H), 7.39–7.36 (m, 4H), 7.29–7.26 (m, 2H), 7.22–7.15 (m, 12H); ^{13}C NMR (126 MHz, $CDCl_3$, ppm): δ = 142.5, 139.7, 134.2, 134.2, 133.7, 129.1, 128.5, 128.4, 127.7, 127.3, 126.6, 125.6.

3. Summary of reported donor number data for solvents and anions

Table 1. DNs for selected solvent [a]

Solvent	DNs for solvent/ kcal mol^{-1}
1,2-dichloroethane	0
dichloromethane	1
acetonitrile	14.1
water	18
tetrahydrofuran	20
N,*N*-dimethylformamide	26.6

[a]Data from ref. 24.

Table 2. DNs for selected anions [a]

Ionic liquid[b]	DNs for anions / kcal mol^{-1}
[emim][PF_6]	−6.2
[emim][TFSI]	7.2
[emim][BF_4]	7.3
[emim][ClO_4]	7.6
[emim][TfO]	20.5

[a]Data from ref. 25. [b][emim] = 1-ethyl-3-methylimidazolium

4. Detailed experimental description of Figure 2

4-1. Voltammetric analysis (Figure 2A)

Cyclic voltammetry measurements were performed in the three-electrode system equipped with Pt disk working electrode (ϕ= 1.6 mm), a Pt plate counter electrode (20 mm × 20 mm) and a SCE reference electrode in 0.1 M supporting electrolyte solution at a scan rate of 100 mVs^{-1}.

Blank cyclic voltammograms were recorded in CH_2Cl_2 containing 0.1 M Bu_4NX in the absence of **PTh-H** under otherwise identical conditions to those used in Fig. 2A. No significant background redox process derived from the supporting electrolytes was observed within the potential window relevant to the oxidation of **PTh-H**, confirming that the voltammetric responses shown in Fig. 2A originate from **PTh-H** and its follow-up reactions.

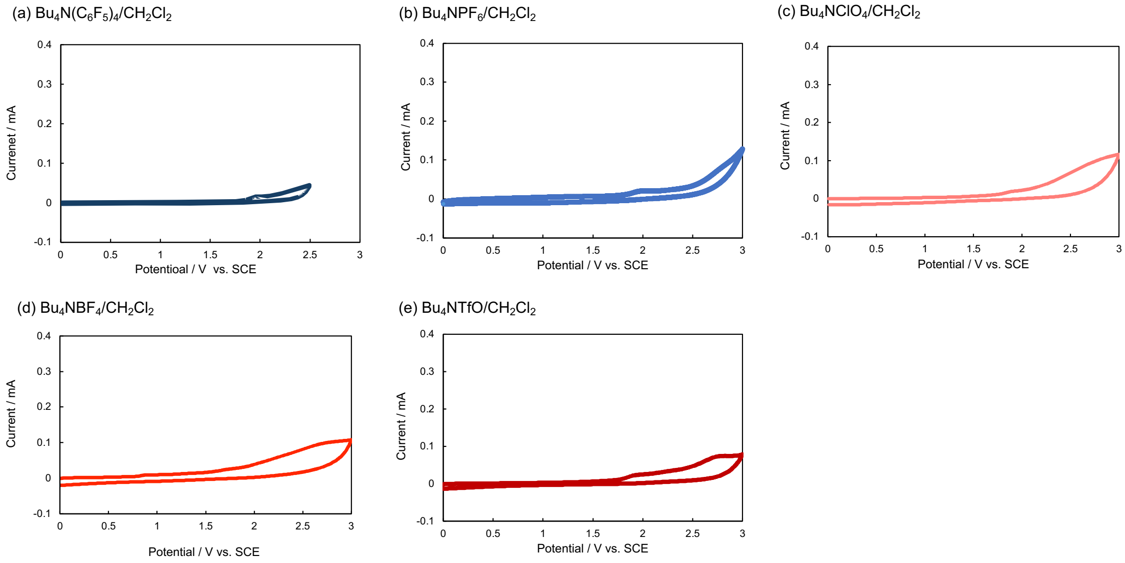

Figure S1. Background CVs measured with 0.1 M Bu_4NX/CH_2Cl_2 electrolyte (X = $B(C_6F_5)_4^-$, PF_6^-, BF_4^-, ClO_4^-, TfO^-) using Pt disk working electrode (ϕ= 1.6 mm), a Pt plate counter electrode (20 mm × 20 mm) and a SCE reference electrode in 0.1 M supporting electrolyte solution at a scan rate of 100 mVs^{-1}.

4-2. Spectroelectrochemistry (Figure 2B)

Spectroelectrochemistry measurements were performed in the three-electrode system equipped with a Pt gauze working electrode, a Pt wire counter electrode, and a SCE reference electrode in thin layer quartz glass spectroelectrochemical cell (1 mm thick) filled with 0.1 M supporting salt electrolyte containing 1 mM of substrates.

4-3. Bulk electrolysis of PTh-H in $Bu_4NB(C_6F_5)_4/CH_2Cl_2$ and Bu_4NTfO/CH_2Cl_2 (Figure 2C)

PTh-H (0.25 mmol, 5 mM) was dissolved in an electrolyte of 0.1 M $Bu_4NB(C_6F_5)_4/CH_2Cl_2$ (20 mL) or Bu_4NTfO/CH_2Cl_2 and used in an anodic chamber. Same electrolyte solution without **PTh-H** was added to a cathodic chamber as an electrolyte. Platinum plates were used as anode (2.5 cm × 4 cm) and cathode (2 cm × 2 cm). The electrolysis was performed by applying a constant current of 10 mA, and 1 F/mol of charge was passed under vigorous stirring (Figure S2). After the electrolysis, an anodic electrolyte solution was collected, and diluted with CH_2Cl_2 for UV-vis and EPR measurements, respectively.

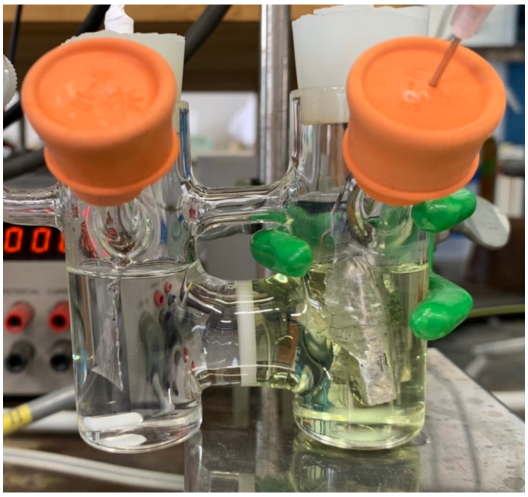

Figure S2. Photograph of electrolytic setup

4-4. Electrolysis of 2,5-diaryl thiophene derivatives, PTh-R (Figure 2D)

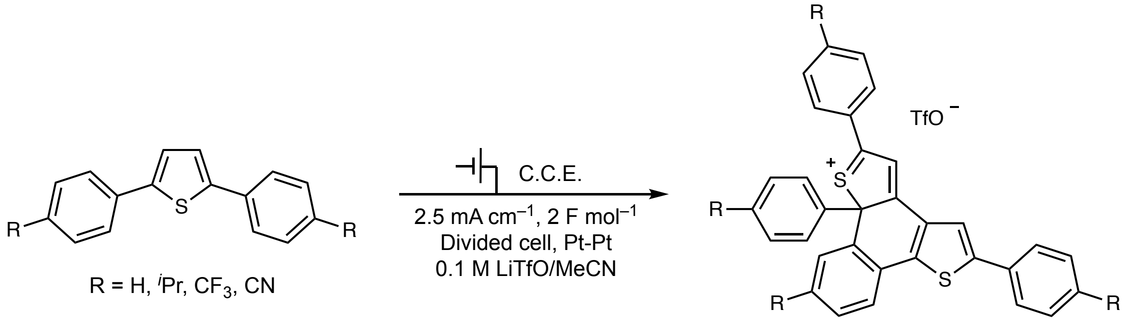

Bulk electrolysis was performed for a series of 2,5-diarylthiophenes. For these experiments, an inorganic electrolyte (LiTfO) was employed to facilitate post-electrolysis workup, and acetonitrile was used as the solvent for this purpose. As demonstrated in the bulk electrolysis experiments described in Section 3–3, comparable synthetic outcomes can also be obtained using Bu_4NTfO/CH_2Cl_2 as an electrolyte. However, under these

conditions, separation of the resulting thiophenium salts from the supporting electrolyte (Bu_4NTfO) proved to be difficult, making this electrolyte–solvent combination less practical for preparative purposes.

PTh-R (R = H, ^{i}Pr, CF_3, CN, OMe; 1.0 mmol) was suspended in an electrolyte of 0.1 M LiTfO/MeCN (20 mL) and used in an anodic chamber. 0.1 M LiTfO/MeCN (20 mL) was added to a cathodic chamber as an electrolyte. Platinum plates were used as anode (2.5 cm × 4 cm) and cathode (2 cm × 2 cm). The electrolysis was performed by applying a constant current of 10 mA, and 2 F/mol of charge was passed under vigorous stirring. After the electrolysis, an anodic electrolyte solution was collected, and the solvent was removed under reduced pressure. The resulting mixture was then extracted with CH_2Cl_2 to obtain a spectroscopically pure product.

Note: Using this procedure, spectroscopically pure thiophenium salts can be obtained. However, gravimetric analysis consistently gives apparent yields exceeding quantitative values, indicating that residual LiTfO electrolyte remains associated with the product. For this reason, isolated yields based on mass are not reported.

[(PTh-H)₂][TfO]: ^{1}H NMR (399.78 MHz, CD_3CN, ppm): δ = 8.36 (s, 1H), 8.25 (d, *J* = 7.8 Hz, 2H), 8.01 (t, *J* = 7.6 Hz, 1H), 7.92 (s, 1H), 7.82 (d, *J* = 7.3 Hz, 2H), 7.77 (t, *J* = 8.0 Hz, 2H), 7.67 (d, *J* = 7.8 Hz, 2H), 7.63-7.60 (m, 2H), 7.45 (d, *J* = 7.3 Hz, 3H), 7.30 (d, *J* = 7.3 Hz, 3H), 7.17 (d, *J* = 8.2 Hz, 2H); ^{13}C NMR (100.53 MHz, CD_3CN, ppm): δ = 209.0, 189.4, 154.6, 148.4, 141.1, 139.0, 138.2, 137.2, 132.8, 132.7, 132.5, 132.3, 131.9, 131.7, 131.3, 130.9, 130.8, 130.5, 130.2, 129.1, 128.4, 127.6, 126.9, 121.6, 84.0, 55.4; ^{19}F NMR (376.46 MHz, CD_3CN, ppm): δ = −77.8 (s, 3F); HRMS (ESI-TOF): calcd for $C_{32}H_{21}S_2^+$ ($[M]^+$) m/z 469.1085, found 469.1086.

[(PTh-^{i}Pr)₂][TfO]: ^{1}H NMR (399.78 MHz, CD_3CN, ppm): δ = 8.34 (s, 1H, Ar), 8.15 (d, *J* = 8.2 Hz, 2H, Ar), 7.83 (s, 1H, Ar), 7.63 (s, 1H, Ar), 7.61 (d, *J* = 7.8 Hz, 2H, Ar), 7.45 (d, *J* = 6.0 Hz, 1H, Ar), 7.40 (d, *J* = 8.2 Hz, 2H, Ar), 7.20 (d, *J* = 7.3 Hz, 1H, Ar), 7.16 (d, *J* = 7.8 Hz, 1H, Ar), 7.10 (d, *J* = 8.7 Hz, 2H, Ar), 6.99 (d, *J* = 8.7 Hz, 2H, Ar), 3.16-2.73 (m, 4H, CH), 1.39 (d, *J* = 6.9 Hz, 6H, CH_3), 1.31 (q, *J* = 3.4 Hz, 6H, CH_3), 1.20 (m, 6H, CH_3), 1.05 (d, *J* = 6.9 Hz, 6H, CH_3); ^{13}C NMR (100.53 MHz, CD_3CN, ppm): δ = 188.5, 165.4, 155.0, 154.5, 152.4, 152.2, 150.2, 148.4, 144.4, 139.9, 137.5, 136.9, 133.8, 133.2, 131.5, 130.8, 130.6, 130.6, 130.2, 130.2, 129.2, 129.0, 128.9, 128.7, 128.0, 127.4, 126.8, 126.6, 125.5, 124.4, 121.4, 121.2, 84.0, 36.5, 35.8, 35.2, 34.9, 24.8, 24.7, 24.6, 24.5, 24.4, 24.0, 24.0; ^{19}F NMR (376.46 MHz, CD_3CN, ppm): δ = −77.9 (s, 3F); HRMS (ESI-TOF): calcd for $C_{44}H_{45}S_2^+$ ($[M]^+$) m/z 637.2957, found 637.2953.

[(PTh-CF$_3$)$_2$][TfO]: ^{1}H NMR (399.78 MHz, CD_3CN, ppm): δ = 8.69 (s, 1H), 8.51 (d, J = 7.3 Hz, 2H), 8.21 (s, 2H), 8.10 (d, J = 8.2 Hz, 2H), 8.05 (d, J = 8.2 Hz, 1H), 7.97 (d, J = 8.2 Hz, 1H), 7.81 (d, J = 8.2 Hz, 2H), 7.66-7.61 (m 4H), 7.40 (d, J = 8.7 Hz, 2H); ^{13}C NMR (100.53 MHz, CD_3CN, ppm): δ = 210.6, 189.6, 153.6, 148.1, 141.42-137.56 (m), 136.34-130.85 (m), 129.64-129.05 (m), 128.93, 128.58-128.02 (m), 127.78-127.59 (m), 127.55, 127.43-127.19 (m), 125.9, 125.7, 125.5, 124.0, 123.60-122.80 (m); ^{19}F NMR (376.46 MHz, CD_3CN, ppm): δ = –61.9 (s, 6F), –62.3 (s, 3F), –63.0 (s, 3F), –77.9 (s, 3F); HRMS (ESI-TOF): calcd for $C_{36}H_{17}F_{12}S_2^+$ ([M]$^+$) m/z 741.0580, found 741.0585.

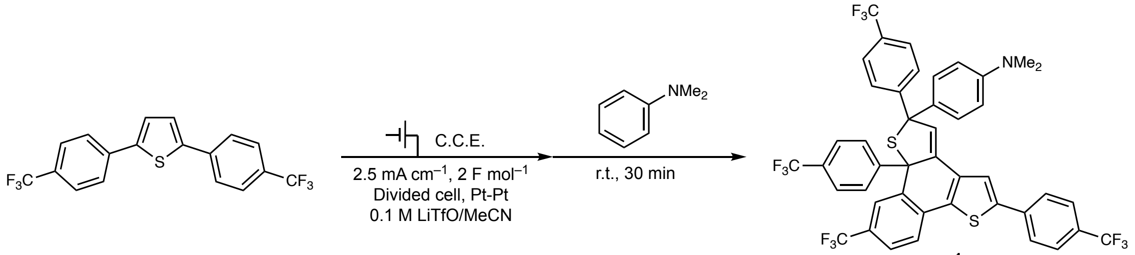

PTh-CF$_3$ (74.5 mg, 0.20 mmol) was suspended in an electrolyte of 0.1 M LiTfO/MeCN (20 mL) and used in an anodic chamber. 0.1 M LiTfO/MeCN (20 mL) was added to a cathodic chamber as an electrolyte. Platinum plates were used as anode (2.5 cm × 4.0 cm) and cathode (2.0 cm × 2.0 cm). Silver wire was used as a reference electrode. The electrolysis was performed by applying a constant current of 10 mA, and 2.0 F/mol of charge was passed under vigorous stirring. After the electrolysis, *N,N*-dimethylaniline (121.2 mg, 1.0 mmol) was added to an anodic solution. The color of the solution immediately changed from deep green to dark yellow. After reacting for 30 min under vigorous stirring, water was added to quench the reaction. The organic layer was washed with water and dried over Na_2SO_4. After filtration and removal of the solvent, the residue was purified by silica gel column chromatography using hexane/dichloromethane (1/4 in vol. ratio) to give **1** as a yellow solid (69 mg, 0.080 mmol, 80%).

1: ^{1}H NMR (399.78 MHz, $CDCl_3$, ppm): δ = 7.92 (s, 1H, Ar), 7.72 (d, J = 8.2 Hz, 2H), 7.65 (d, J = 8.7 Hz, 2H), 7.62 (d, J = 8.2 Hz, 1H, Ar), 7.55–7.51 (m, 6H, Ar), 7.25 (s, 2H, Ar), 7.07 (d, J = 8.7 Hz, 2H, Ar), 7.02 (d, J = 8.7 Hz, 2H, Ar), 6.68 (s, 1H, Ar), 6.57 (d, J = 8.7 Hz, 2H, Ar), 2.89 (s, 6H, CH_3); ^{13}C{^{19}F} NMR (126 MHz, $CDCl_3$, ppm): δ = 149.8, 148.4, 148.2, 144.2, 139.3, 138.1, 137.3, 136.7, 135.4, 134.4, 132.5, 131.9, 130.4, 129.6, 129.5, 129.3, 128.8, 128.6, 127.0, 126.3, 126.1, 125.6, 125.4, 125.2, 125.2, 124.4, 124.2, 124.1, 124.0, 124.0, 122.2, 112.2, 72.7, 69.4, 40.5; ^{19}F NMR (376.46 MHz, $CDCl_3$,

ppm): δ = -62.70 (s, 3F), -62.88 (s, 3F), -63.06 (s, 3F), -63.13 (s, 3F) ; HRMS (ESI-TOF): calcd for $C_{44}H_{27}F_{12}NNaS_2^+$ ($[M+Na]^+$) m/z 884.1291, found 884.1306.

4-5. Electrolysis and voltammetric analysis of dimeric precursor (Figure 2E)

bis(PTh-H) (0.25 mmol) was dissolved in an electrolyte of 0.1 M of Bu_4NTfO/CH_2Cl_2 or $Bu_4NB(C_6F_5)_4/CH_2Cl_2$ (20 mL) and used in an anodic chamber. Same electrolyte solution without **PTh-H** was added to a cathodic chamber as an electrolyte. Platinum plates were used as anode (2.5 cm × 4 cm) and cathode (2 cm × 2 cm). The electrolysis was performed by applying a constant current of 10 mA, and 2 F/mol of charge was passed under vigorous stirring. After the electrolysis, an anodic electrolyte solution was collected, and the solvent was removed under reduced pressure. CD_3CN was added to the crude mixture, and subjected to ^1H NMR measurements. Formation of thiophenium cation was observed for both cases.

On the other hand, CV data of **bis(PTh-H)** under coordinating and weakly coordinating conditions showed significant difference as discussed in the manuscript, where fully irreversible voltammetry was observed only under coordinating condition. These results suggest that the oxidative cyclization proceeds both under coordinating and weakly-coordinating conditions, while the reaction under weakly-coordinating condition is relatively slow.

Cyclic voltammetry of **bis(PTh-H)** was performed in CH_2Cl_2 containing 0.1 M $Bu_4NB(C_6F_5)_4$ at different scan rates to evaluate whether the observed redox response involves significant surface adsorption. The measurements were conducted using a three-electrode system equipped with a Pt disk working electrode, a Pt plate counter electrode, and an SCE reference electrode. The scan rates were varied from 10 to 1000 mV s^{-1}. As shown in Figure S3a, **bis(PTh-H)** exhibited a reversible oxidation response under these weakly coordinating electrolyte conditions. The anodic peak current increased with increasing scan rate. A plot of the anodic peak current against the square root of the scan rate showed a linear relationship (Figure S3b), indicating that the oxidation of **bis(PTh-H)** is governed by a diffusion-controlled electron-transfer process. This result suggests that the redox behavior of **bis(PTh-H)** under these conditions does not involve significant surface adsorption on the Pt electrode.

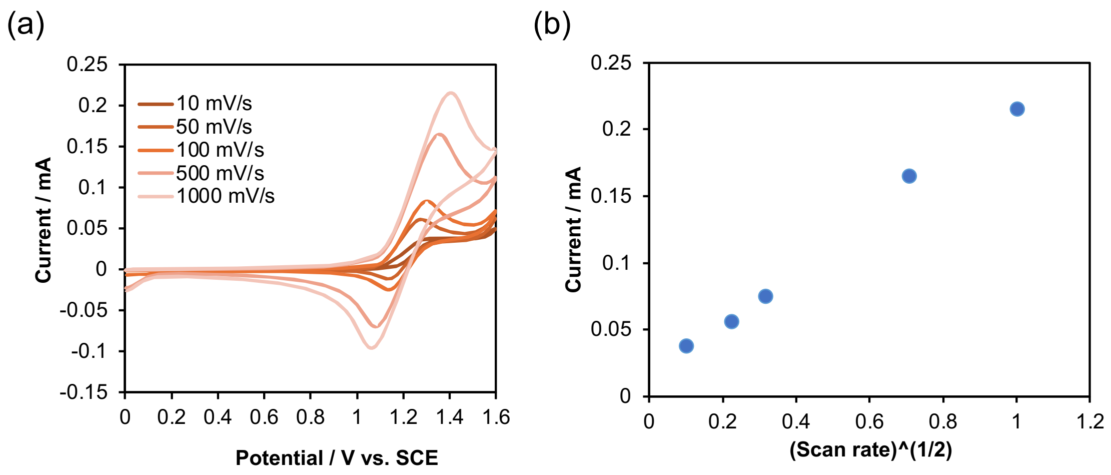

Figure S3. (a) Cyclic voltammograms of bis(PTh-H) recorded in CH_2Cl_2 containing 0.1 M $Bu_4NB(C_6F_5)_4$ at different scan rates: 10, 50, 100, 500, and 1000 mV s^{-1}. (b) Plot of the anodic peak current against the square root of the scan rate. The linear relationship indicates that the oxidation of bis(PTh-H) is governed by a diffusion-controlled electron-transfer process rather than significant surface adsorption on the Pt electrode.

5. Detailed experimental description of Figure 3

*5-1. Electrolysis of 6-methoxy-2-(4-methoxyphenyl)benzo[b]thiophene **(BTh)** (Figure 3A)*

5-1-1. Electrolysis of BTh in coordinating electrolyte (1 F mol^{-1})

BTh (1.0 mmol) was suspended in an electrolyte of 0.1 M LiTfO/MeCN (20 mL) and used in an anodic chamber. 0.1 M LiTfO/MeCN (20 mL) was added to a cathodic chamber as an electrolyte. Platinum plates were used as anode (2 cm × 2 cm) and cathode (2 cm × 2 cm). The electrolysis was performed by applying a constant current of 4 mA, and 1 F/mol of charge was passed under vigorous stirring. After the electrolysis, an anodic electrolyte solution was collected, and the solvent was removed under reduced pressure. The yield of dimeric product **bis(BTh)** was determined by ^{1}H NMR.

bis(BTh): ^{1}H NMR (500 MHz, CD_3CN, ppm): δ = 3.70 (s, 3H), 3.84 (s, 3H), 6.76-6.68 (m, 2H), 6.81 (dd, *J* = 8.8, 2.4 Hz, 1H), 7.02 (d, *J* = 8.8 Hz, 1H), 7.24-7.15 (m, 2H), 7.49 (d, *J* = 2.4 Hz, 1H); ^{13}C NMR (500 MHz, CD_3CN, ppm): δ = 163.72, 159.51, 157.91, 140.92, 140.02, 138.81, 134.62, 129.08, 123.54, 114.43, 114.04, 105.10, 55.37, 55.00; HRMS (ESI-TOF): calcd for $C_{32}H_{27}O_4S_2^+$ ([H]+) m/z 539.1454, found 539.1335.

5-1-2. Electrolysis of BTh in coordinating electrolyte (2 F mol^{-1})

BTh (1.0 mmol) was suspended in an electrolyte of 0.1 M LiTfO/MeCN (20 mL) and used in an anodic chamber. 0.1 M LiTfO/MeCN (20 mL) was added to a cathodic chamber as an electrolyte. Platinum plates were used as anode (2 cm × 2 cm) and cathode (2 cm × 2 cm). The electrolysis was performed by applying a constant current of 4 mA, and 2 F/mol of charge was passed under vigorous stirring. After the electrolysis, an anodic electrolyte solution was collected, and the solvent was removed under reduced pressure. The resulting mixture was then extracted with CH_2Cl_2 and washed with hexane to obtain a spectroscopically pure product, **[BTh$_2$][TfO]**.

[BTh$_2$][TfO]: ^{1}H NMR (500MHz, CD_3CN, ppm): δ = 8.40 (d, J = 9.6 Hz, 1H), 8.06 (d, J = 8.9 Hz, 1H), 7.84 (d, J = 8.6 Hz, 1H), 7.50 (d, J = 2.4 Hz, 1H), 7.50 (d, J = 2.3 Hz, 1H), 7.25 (d, J = 2.4 Hz, 1H), 7.16 (dd, J = 8.9, 2.4 Hz, 1H), 7.10 (dd, J = 8.9, 2.7 Hz, 1H), 7.09 (dd, J = 9.6, 2.4 Hz, 1H), 6.97 (d, J = 9.2 Hz, 2H), 4.17 (s, 3H), 3.98 (s, 3H), 3.88 (s, 3H), 3.65 (s, 3H); ^{13}C NMR (500 MHz, CD_3CN, ppm): δ = 176.56, 175.26, 141.45, 136.10, 132.27, 128.24, 125.01, 123.08, 116.11, 115.59, 108.55, 107.86, 59.60, 57.18, 56.62, 55.98; HRMS (ESI-TOF): calcd for $C_{32}H_{25}NaO_4S_2^+$ ([M]+) m/z 537.1189, found 537.1189.

5-1-3. Electrolysis of BTh in weakly coordinating electrolyte

BTh (0.05 mmol) was dissolved in an electrolyte of 0.1 M $Bu_4NB(C_6F_5)_4/CH_2Cl_2$ (10 mL) and used in an anodic chamber. 0.1 M $Bu_4NB(C_6F_5)_4/CH_2Cl_2$ (10 mL) was added to a cathodic chamber as an electrolyte. Platinum plates were used as anode (2 cm × 2 cm) and cathode (2 cm × 2 cm). The electrolysis was performed by applying a constant current of 4 mA, and 1 F/mol of charge was passed under vigorous stirring. After the electrolysis, deep green color solution was obtained (Figure S4a). The resulting solution was subjected to EPR measurement to show a signal was observed, suggesting the formation of persistent radical cation species, **BTh**$^{\bullet+}$ (Figure S4b).

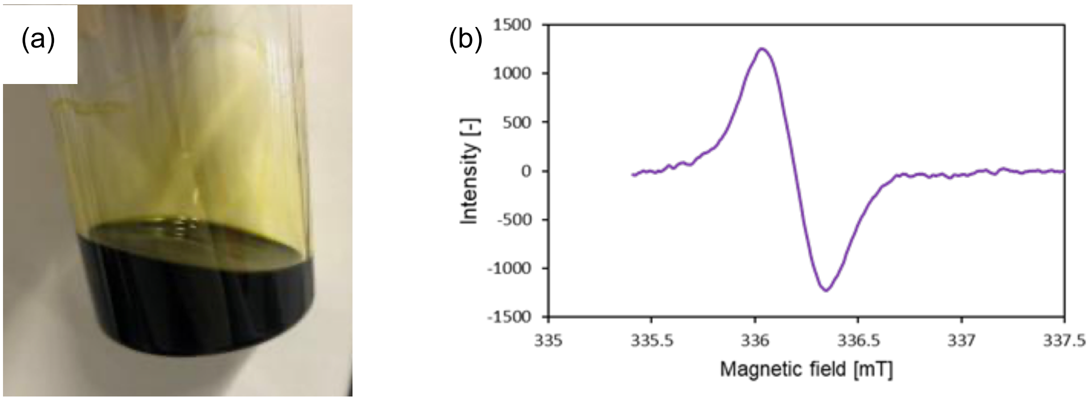

Figure S4. (a) EPR spectrum of **BTh**$^{\bullet+}$ after electrolysis under weakly coordinating conditions. (b) Photograph of reaction solutions after the electrolysis.

5-2. Electrolysis of ***BTh*** *in various electrolyte (Figure 3B)*

BTh (0.05 mmol) was suspended in an electrolyte of 0.1 M Bu_4NX/CH_2Cl_2 (20 mL, X = TfO^-, ClO_4^-, BF_4^-, $B(C_6F_5)_4^-$) or 0.1 M Bu_4NX/MeCN (10 mL, X = TfO^-, $B(C_6F_5)_4^-$) and used in an anodic chamber. 0.1 M LiTfO/MeCN (10 mL) was added to a cathodic chamber as an electrolyte. Platinum plates were used as anode (2 cm × 2 cm) and cathode (2 cm × 2 cm). The electrolysis was performed by applying a constant current of 4 mA, and 1 F/mol of charge was passed under vigorous stirring. After the electrolysis, an anodic electrolyte solution was collected, and the solvent was removed under reduced pressure. The yield of dimeric product **bis(BTh)** was determined by ^{1}H NMR.

5-3. Addition of TfO^- to accumulated ***BTh***$^{\bullet+}$ *(Figure 3C)*

BTh (0.05 mmol) was dissolved in an electrolyte of 0.1 M $Bu_4NB(C_6F_5)_4/CH_2Cl_2$ (10 mL) and used in an anodic chamber. 0.1 M $Bu_4NB(C_6F_5)_4/CH_2Cl_2$ (10 mL) was added to a cathodic chamber as an electrolyte. Platinum plates were used as anode (2 cm × 2 cm) and cathode (2 cm × 2 cm). The electrolysis was performed by applying a constant current of 4 mA, and 1 F/mol of charge was passed under vigorous stirring. After the electrolysis, Bu_4NTfO was added to the reaction mixture. The deep green color of reaction solution immediately change to dark brown, indicating the smooth progress of chemical reaction. After reacting for 1 h under vigorous stirring, the solvent was removed under reduced pressure, and placed by silica-gel column chromatography using hexane/dichloromethane mixed solvent as eluent to afford **bis(BTh)** in 91% yield.

6. Single-crystal X-ray diffraction measurement of 1

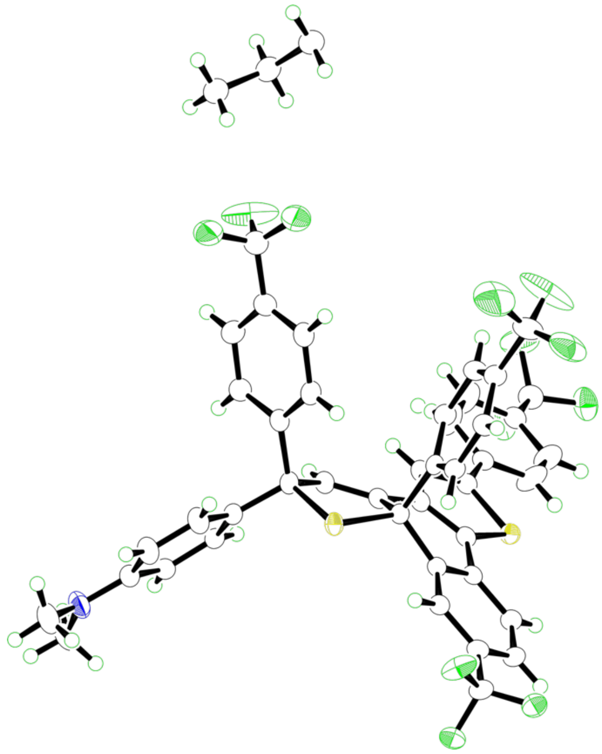

Figure S5. X-ray crystal structure of **1・0.5(C_6H_{14})**

Crystallographic data are available free of charge from the Cambridge Crystallographic Data Centre (https://summary.ccdc.cam.ac.uk/structure-summary-form under deposition number 2526613).

Table S1. Crystallographic data of **1**.

**Compounds**	**1・0.5(C_6H_{14})**
Solvent system	$CHCl_3$/hexane
Empirical formula	$C_{47}H_{34}F_{12}NS_2$
Formula weight	904.87
Temperature/K	293
Crystal system	triclinic
Space group	P-1
a/Å	9.46110(10)
b/Å	11.4919(2)
c/Å	19.2439(3)
α/°	101.6550(10)
β/°	94.1460(10)
γ/°	93.3850(10)
Volume/Å^3	2037.99(5)
Z	2
ρ_{calc} g/cm^3	1.475
μ/mm^{-1}	1.996
Color and shape	colorless plate
Reflections	27358
R_{int}	0.0412
Data/restraints/parameters	8179/0/562
GOF	1.062
R_1 [I>=2σ(I)]	0.0556
wR_2 [I>=2σ(I)]	0.1535
R_1 [all data]	0.0597
wR_2 [all data]	0.1580
Largest diff.peak/hole/e Å^{-3}	1.31/-0.79
Solvent mask	None
CCDC No.	2526613

7. Supporting References

1. S. Li, F. Liu, C. Qian, S. Zhou, *ChemistrySelect*, **2023**, 8, e202302972.

2. G. M. Sheldrick, Crystal Structure Refinement with SHELXL. *Acta Crystallogr C Struct Chem* **2015**, *71* (1). https://doi.org/10.1107/S2053229614024218, 3-8.

3. G. M. Sheldrick, SHELXT - Integrated Space-Group and Crystal-Structure Determination. *Acta Crystallogr A Found Adv* **2015**, *71* (1). https://doi.org/10.1107/S2053273314026370, 3-8.

8. NMR charts

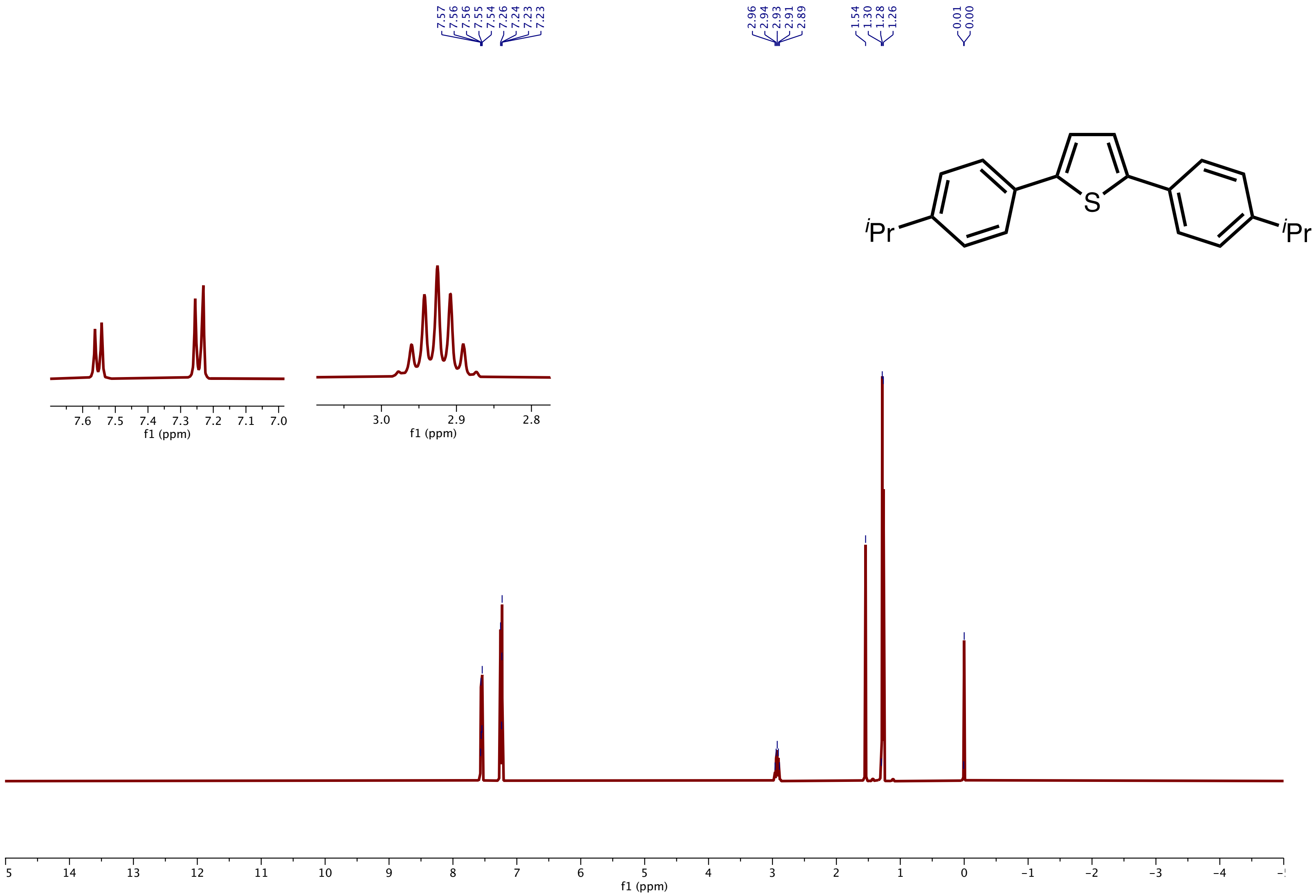

^{1}H NMR spectrum (399.78 MHz, $CDCl_3$, 25 °C) of **PTh-^{i}Pr**.

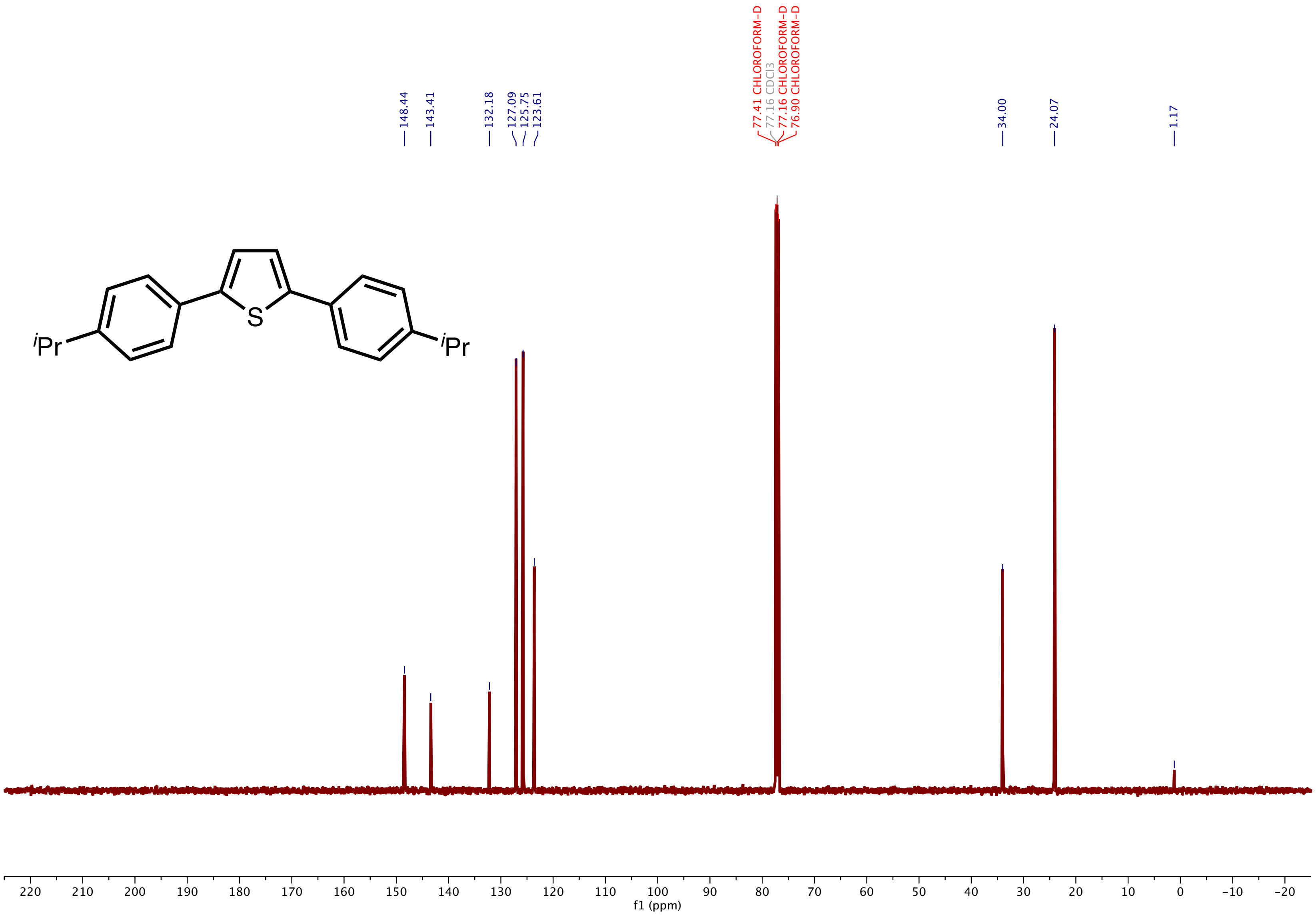

^{13}C NMR spectrum (100.53 MHz, $CDCl_3$, 25 °C) of **PTh-*i*Pr**.

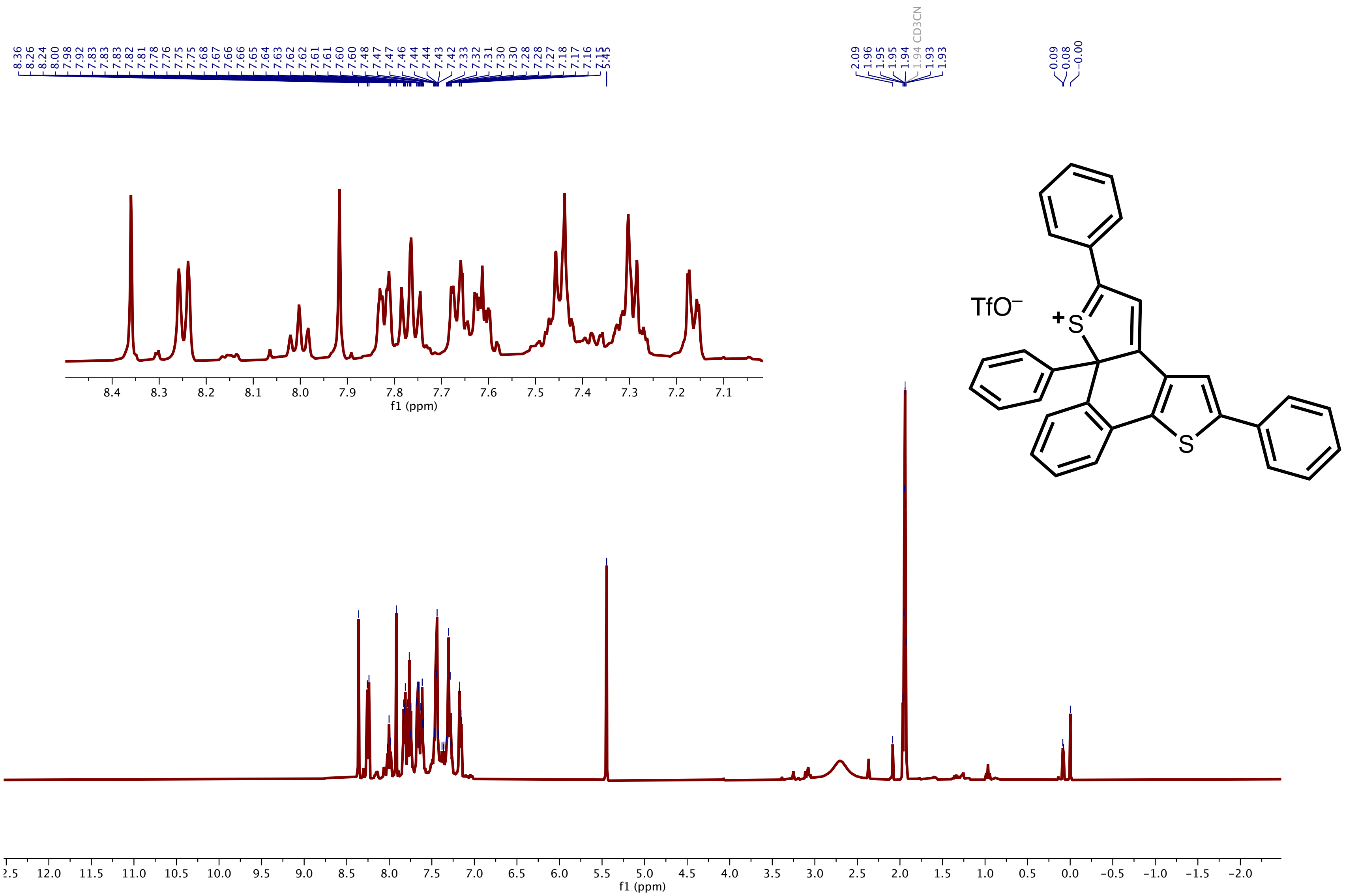

^{1}H NMR spectrum (399.78 MHz, $CDCl_3$, 25 °C) of **[(PTh-H)$_2$][TfO]**.

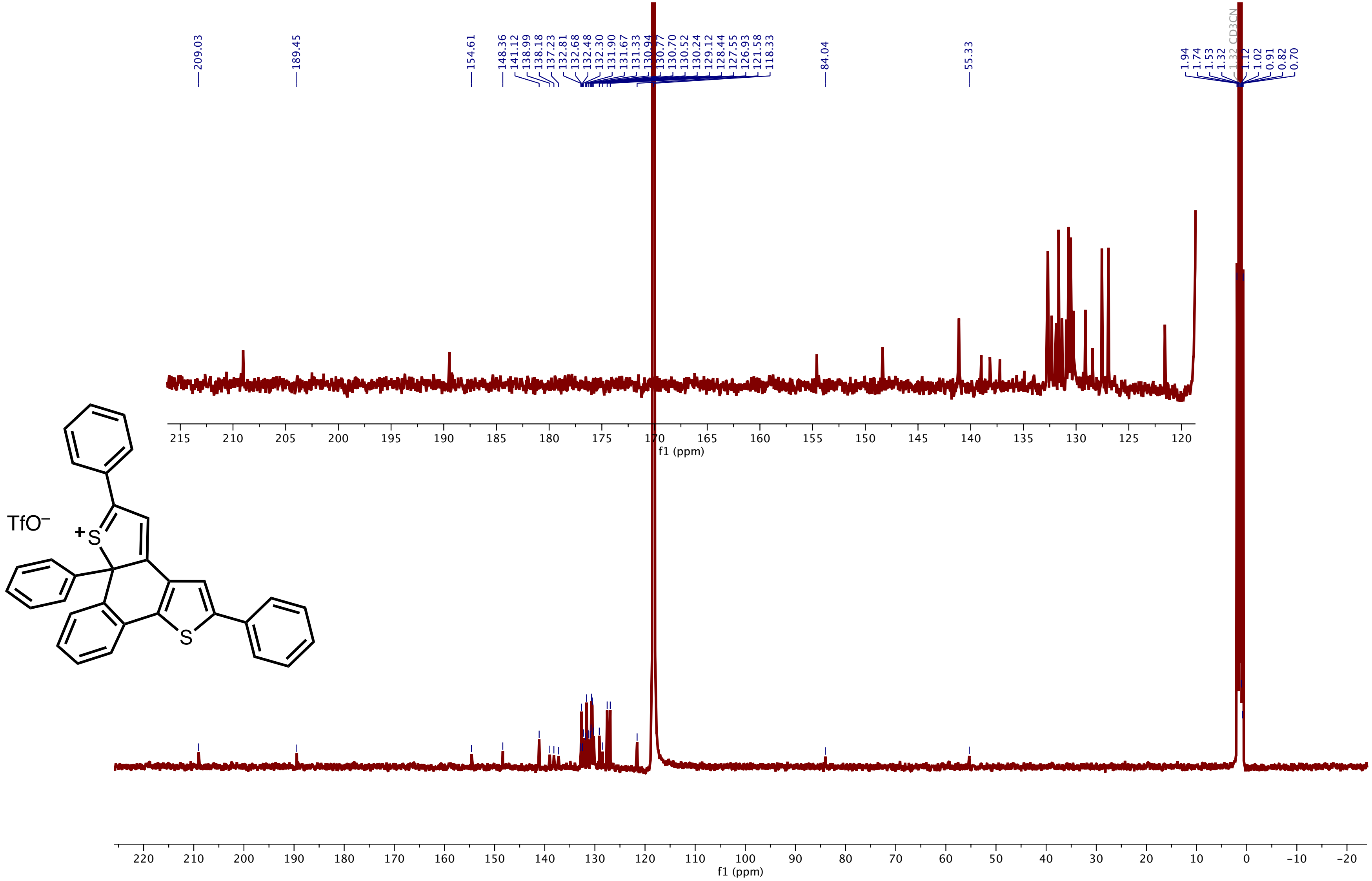

^{13}C NMR spectrum (100.53 MHz, $CDCl_3$, 25 °C) of **[(PTh-H)$_2$][TfO]**.

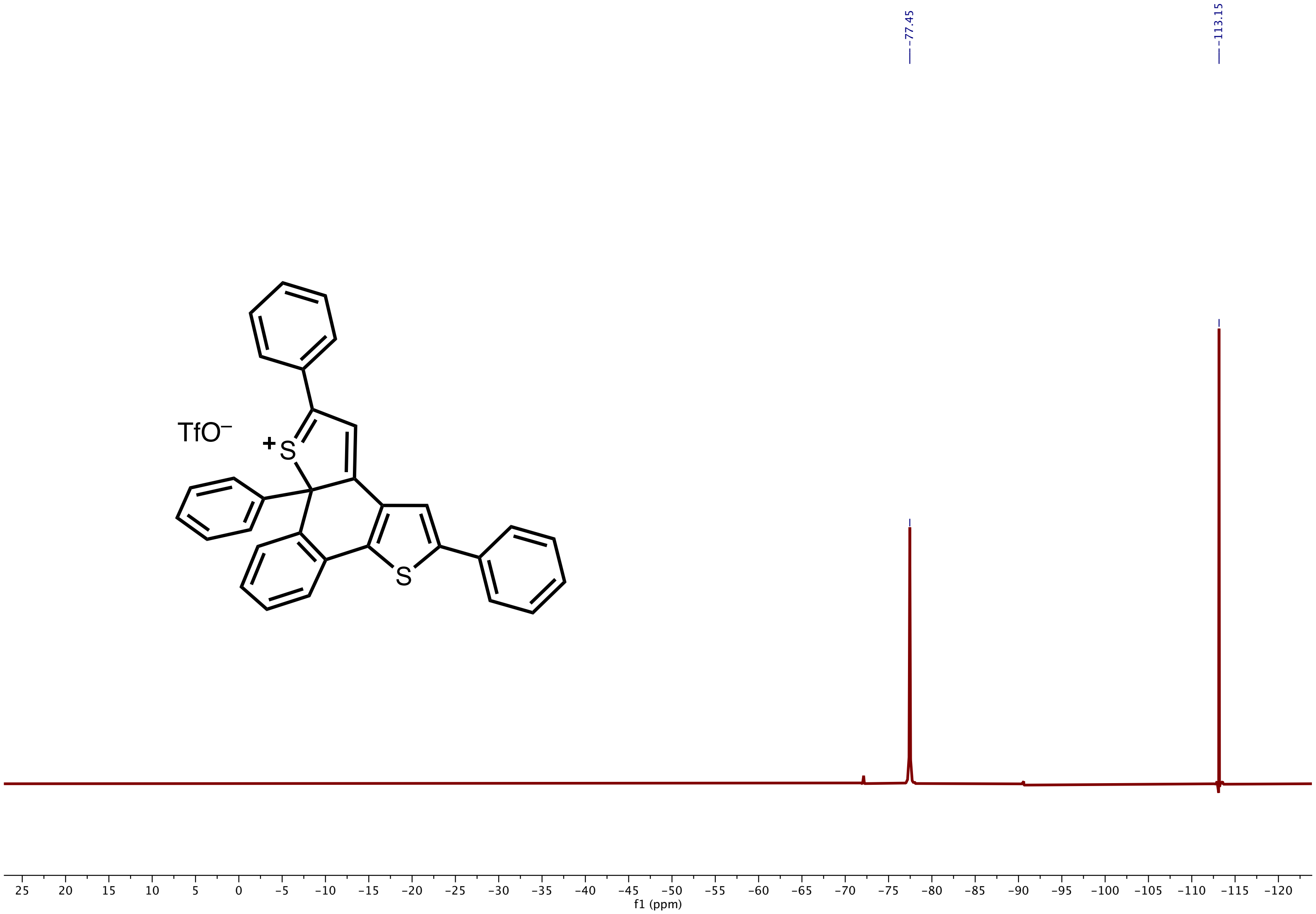

Figure 2-5-24. ^{19}F NMR spectrum (376.46MHz, CD_3CN, 25 °C) of **[(PTh-H)$_2$][TfO]**.

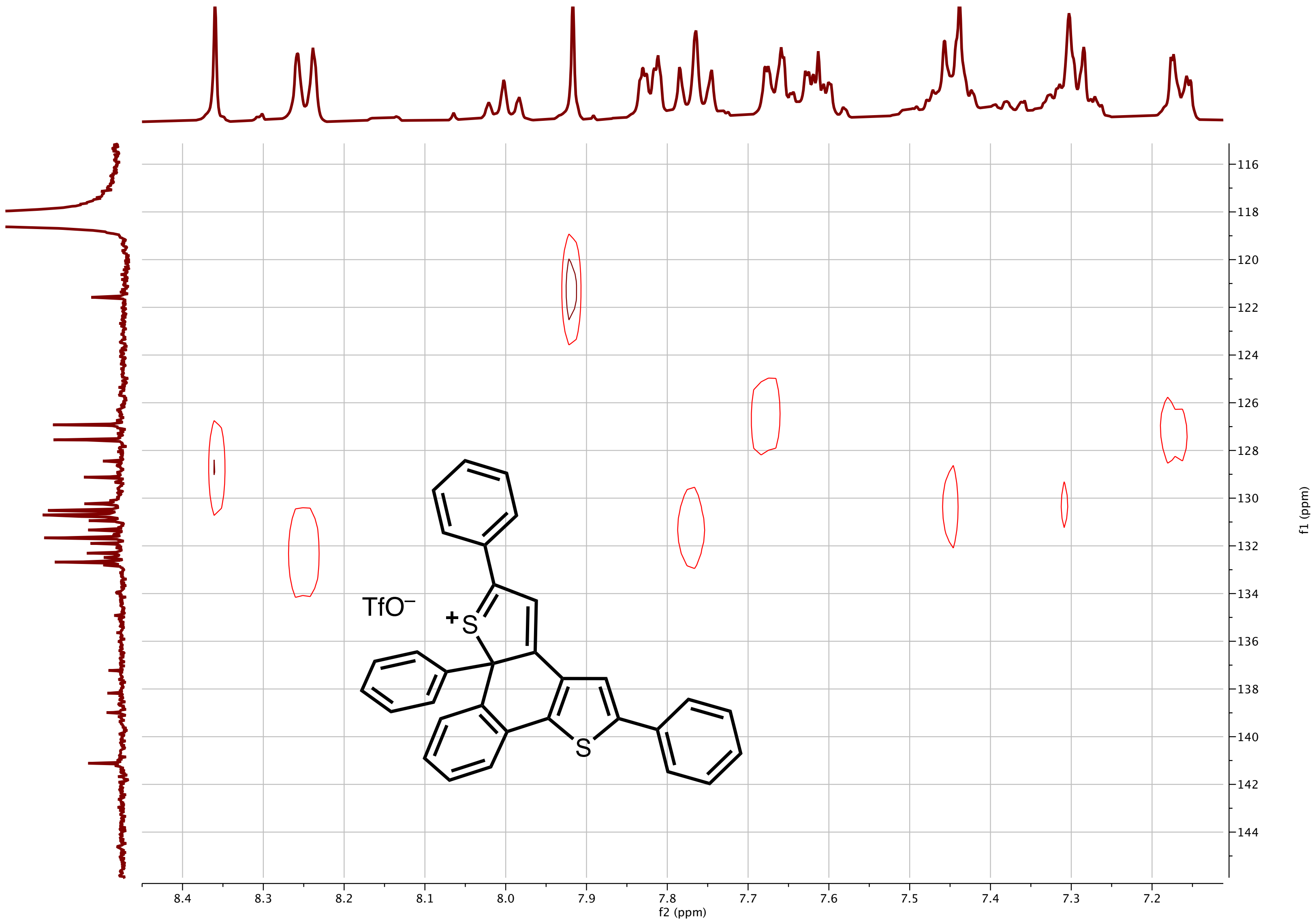

HMQC (CD_3CN, 25 °C) of **[(PTh-H)$_2$][TfO]** (enlarged at the aromatic region of ^{1}H NMR)

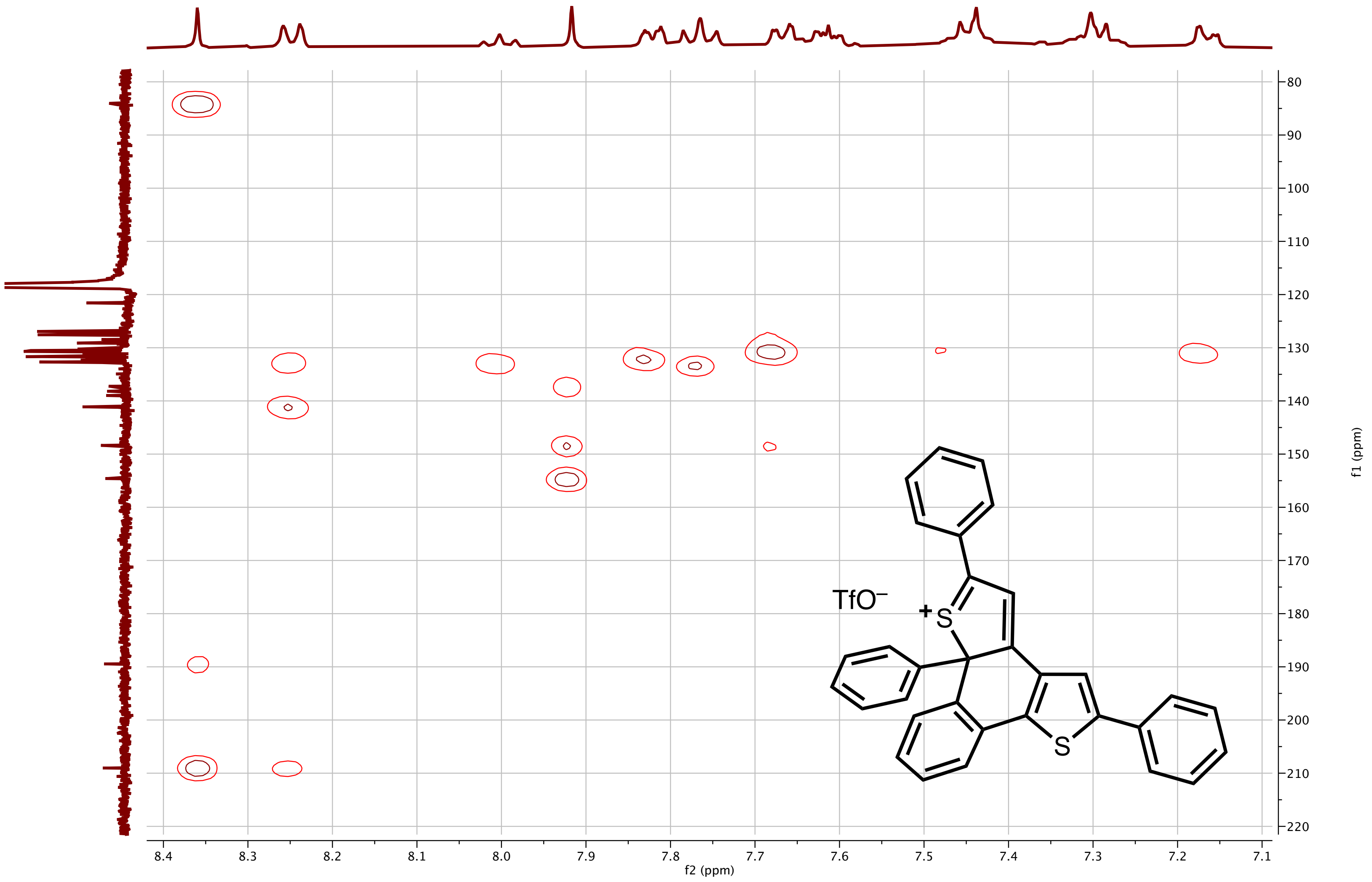

HMBC (CD_3CN, 25 °C) of **[(PTh-H)$_2$][TfO]** (enlarged at the aromatic region of ^{1}H NMR)

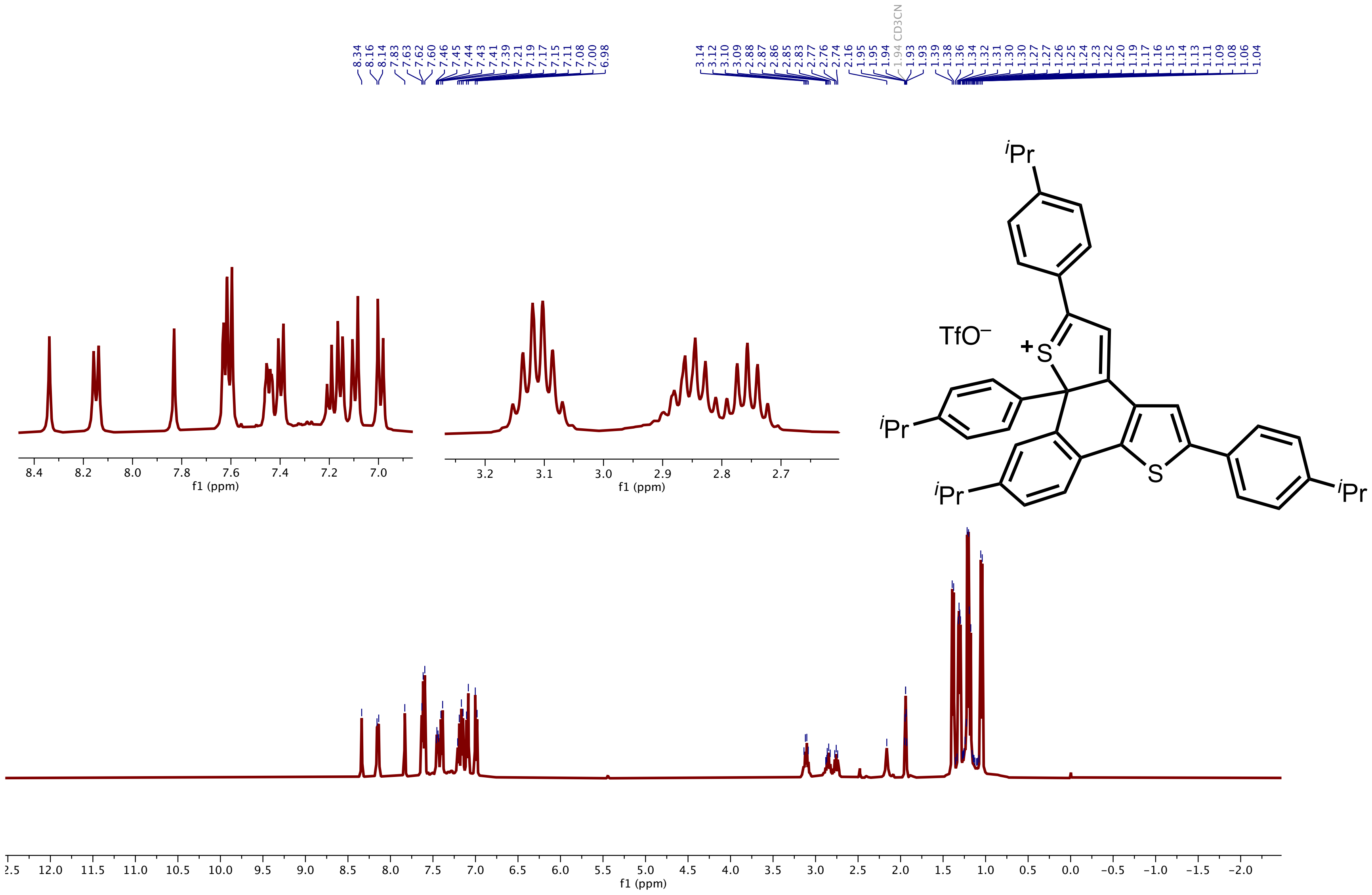

^{1}H NMR spectrum (^{1}H:399.78 MHz, CD_3CN, 25 °C) of **[(PTh-^{i}Pr)$_2$][TfO]**.

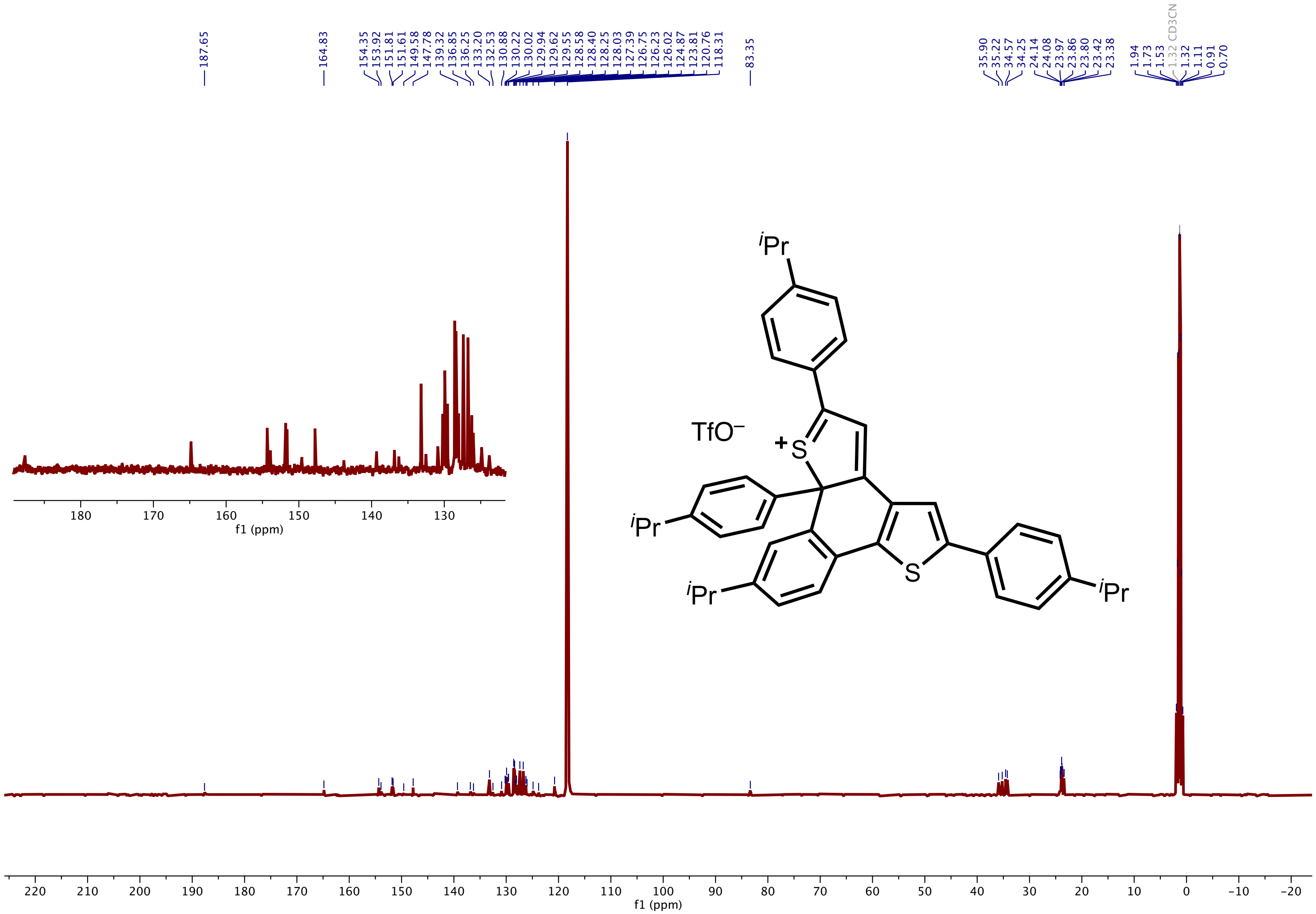

^{13}C NMR spectrum (^{13}C: 100.53 MHz, CD_3CN, 25 °C) spectrum of **[(PTh-^{i}Pr)$_2$][TfO]**.

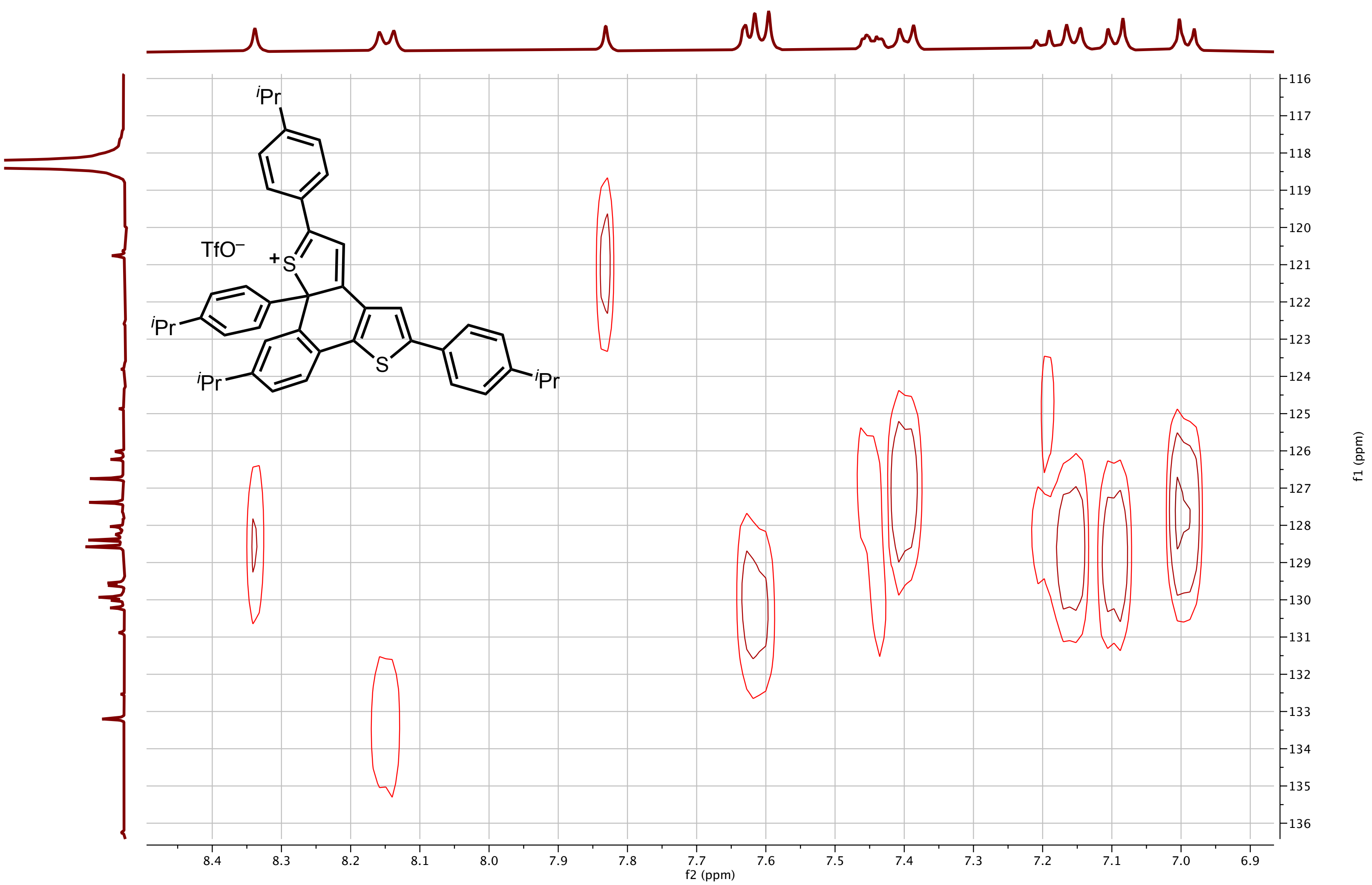

HMQC (CD_3CN, 25 °C) of **[(PTh-^{i}Pr)$_2$][TfO]** (enlarged at the aromatic region of ^{1}H NMR)

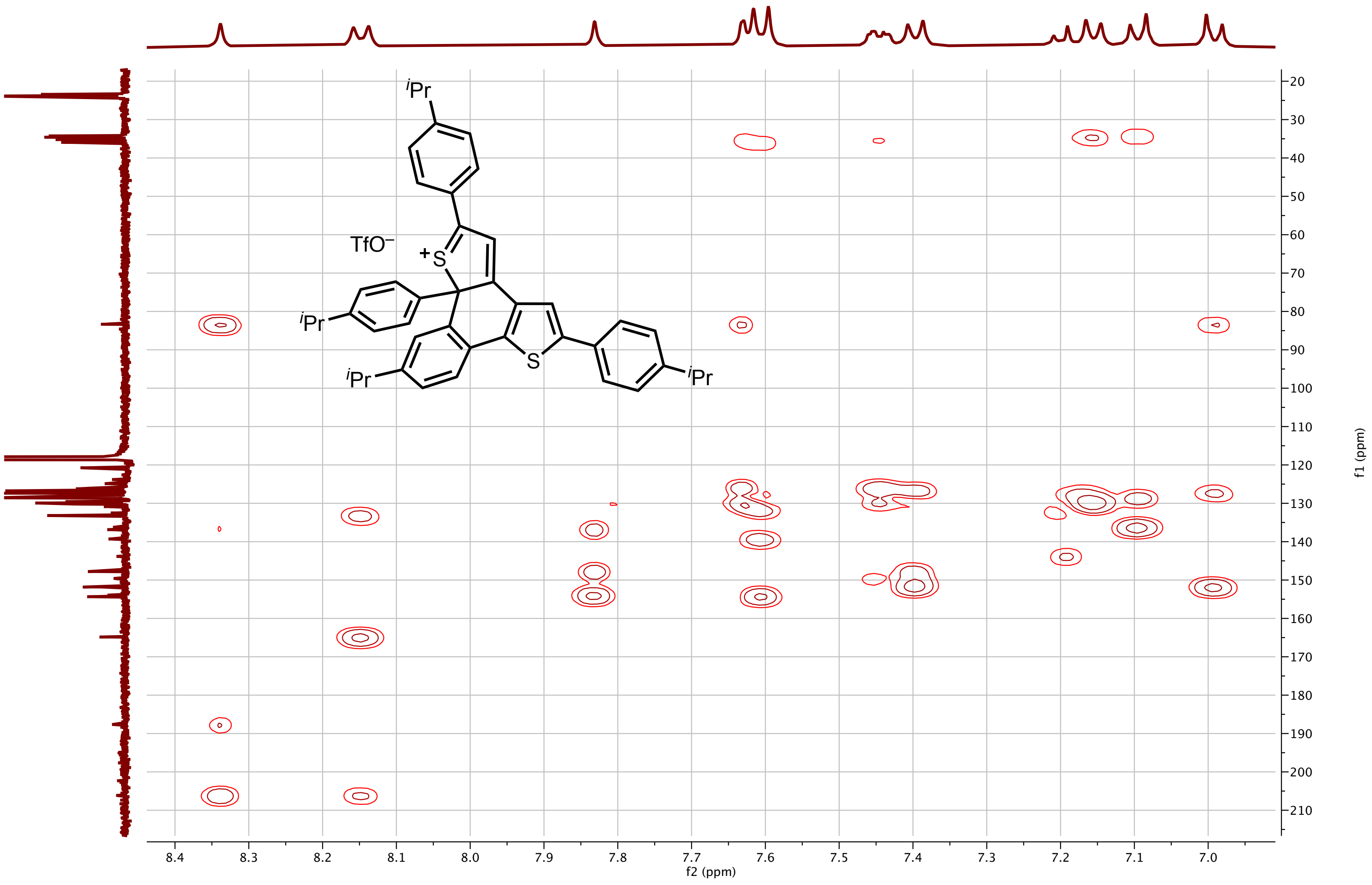

HMBC (CD_3CN, 25 °C) of **[(PTh-^{i}Pr)$_2$][TfO]** (enlarged at the aromatic region of ^{1}H NMR)

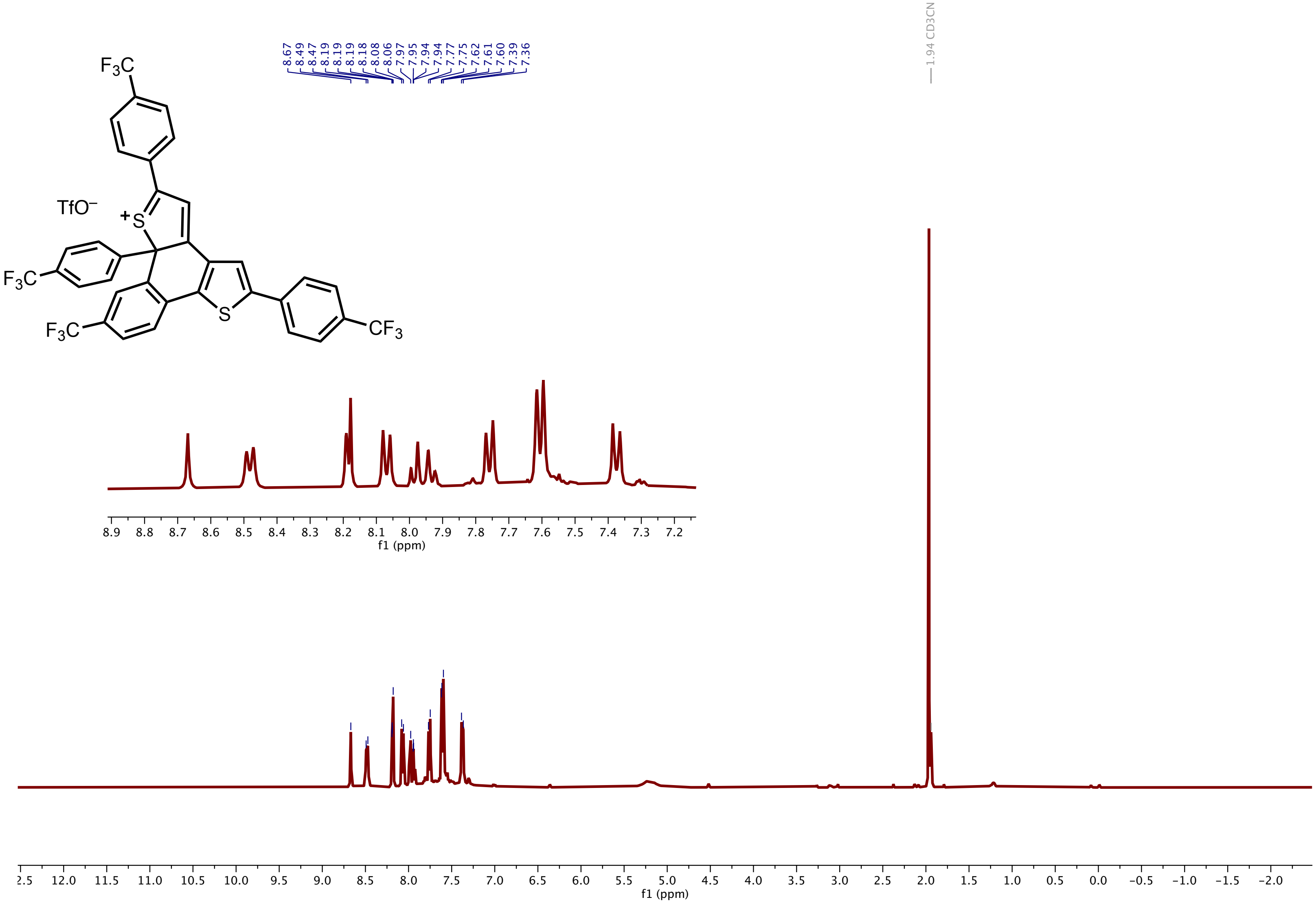

^{1}H NMR spectrum (^{1}H:399.78 MHz, CD_3CN, 25 °C) of **[(PTh-CF$_3$)$_2$][TfO]**.

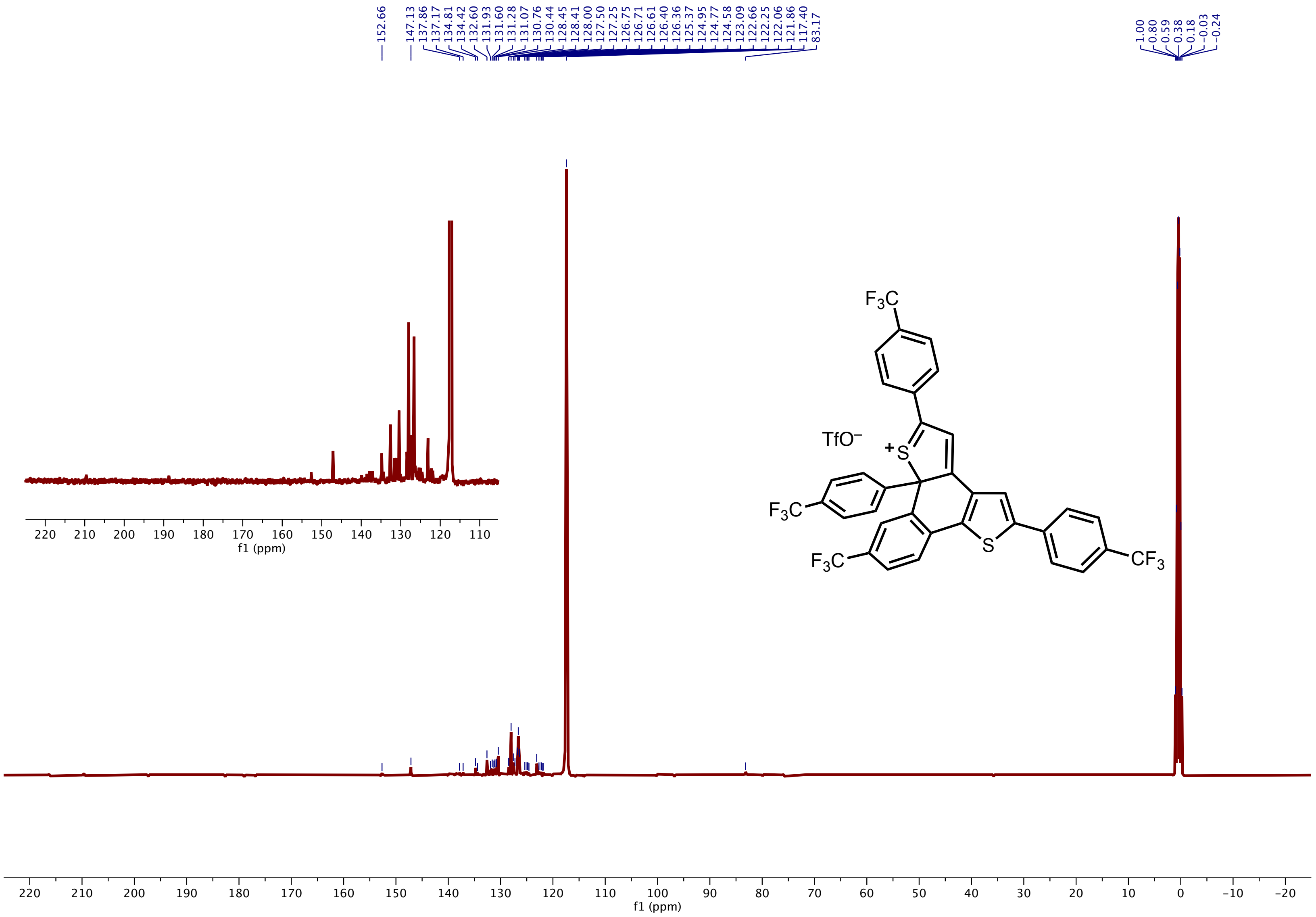

^{13}C NMR spectrum (^{13}C: 100.53 MHz, CD_3CN, 25 °C) spectrum of **[(PTh-CF$_3$)$_2$][TfO]**.

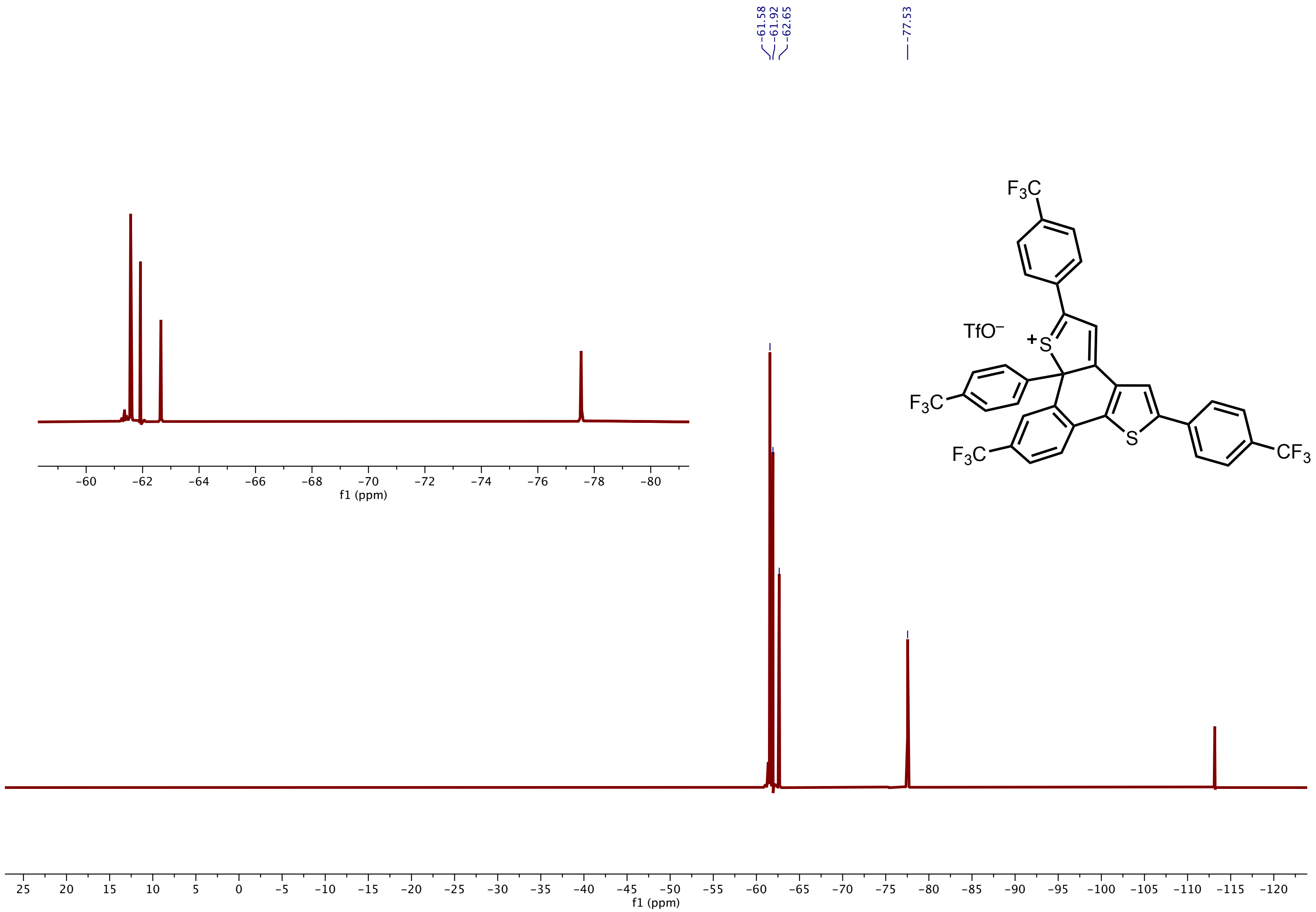

^{19}F NMR spectrum (^{19}F: 376.46 MHz, CD_3CN, 25 °C) spectrum of **[(PTh-CF$_3$)$_2$][TfO]**.

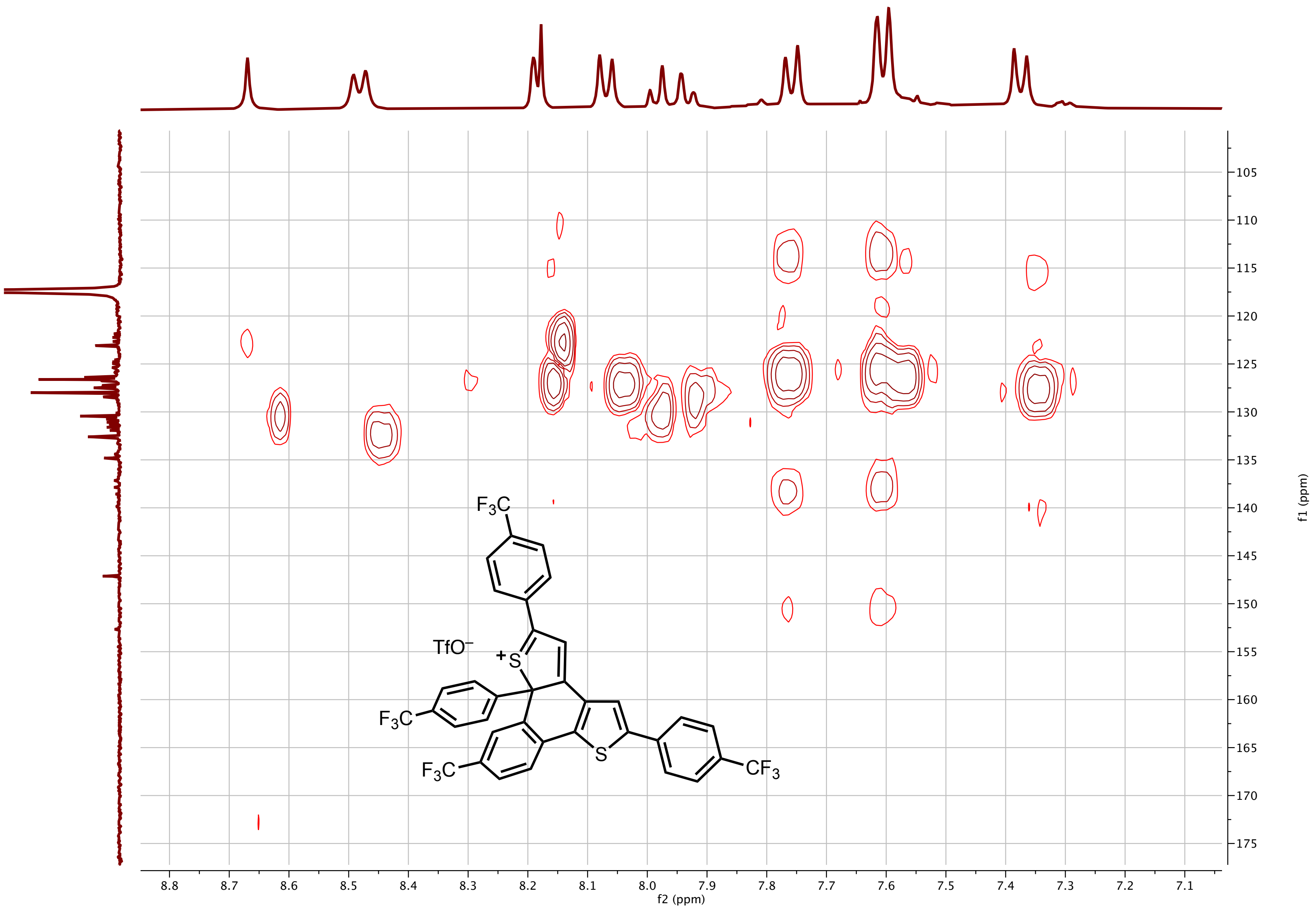

HMQC (CD_3CN, 25 °C) of **[(PTh-CF$_3$)$_2$][TfO]** (enlarged at the aromatic region of ^{1}H NMR)

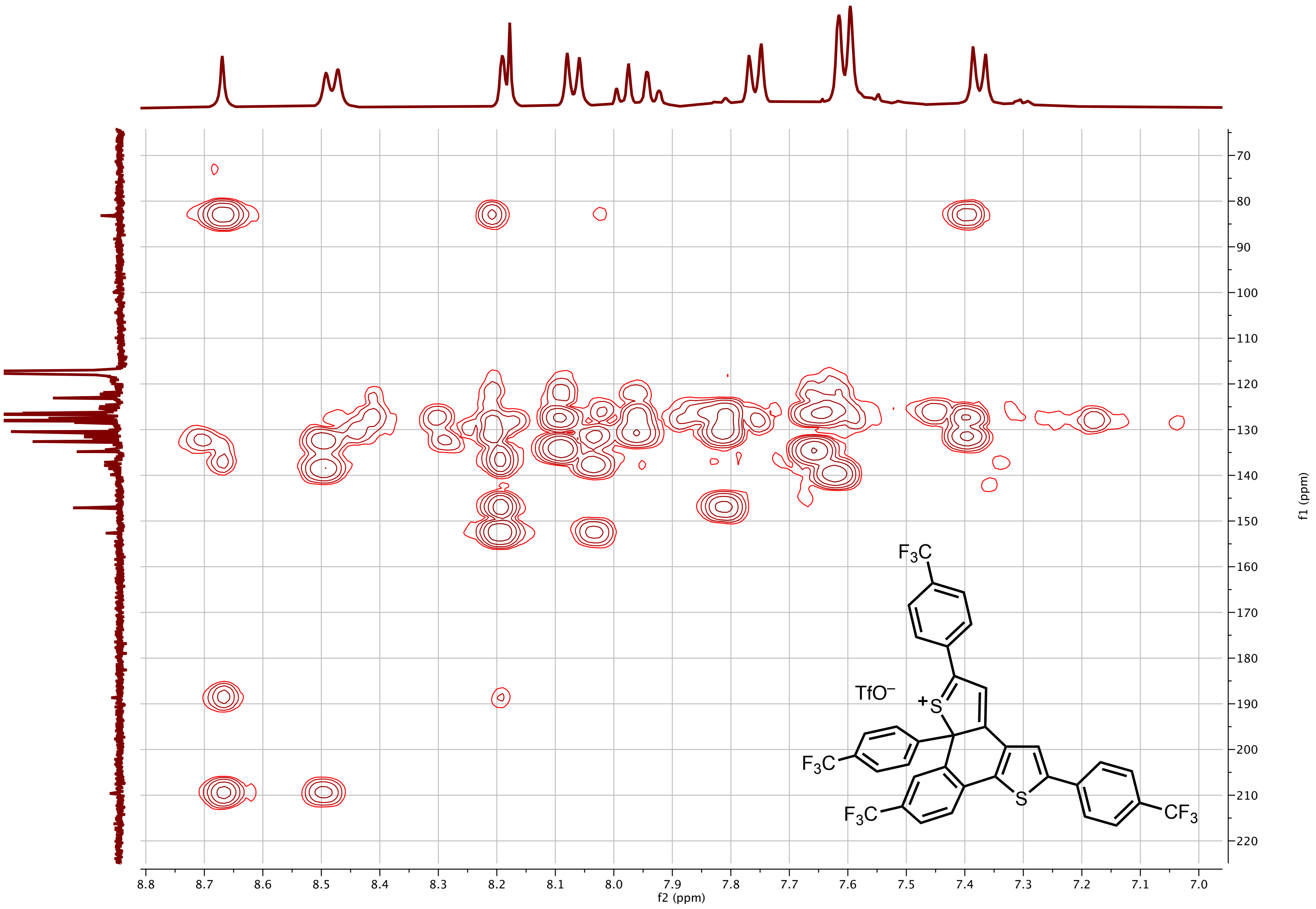

HMBC (CD_3CN, 25 °C) of **[(PTh-CF$_3$)$_2$][TfO]** (enlarged at the aromatic region of ^{1}H NMR)

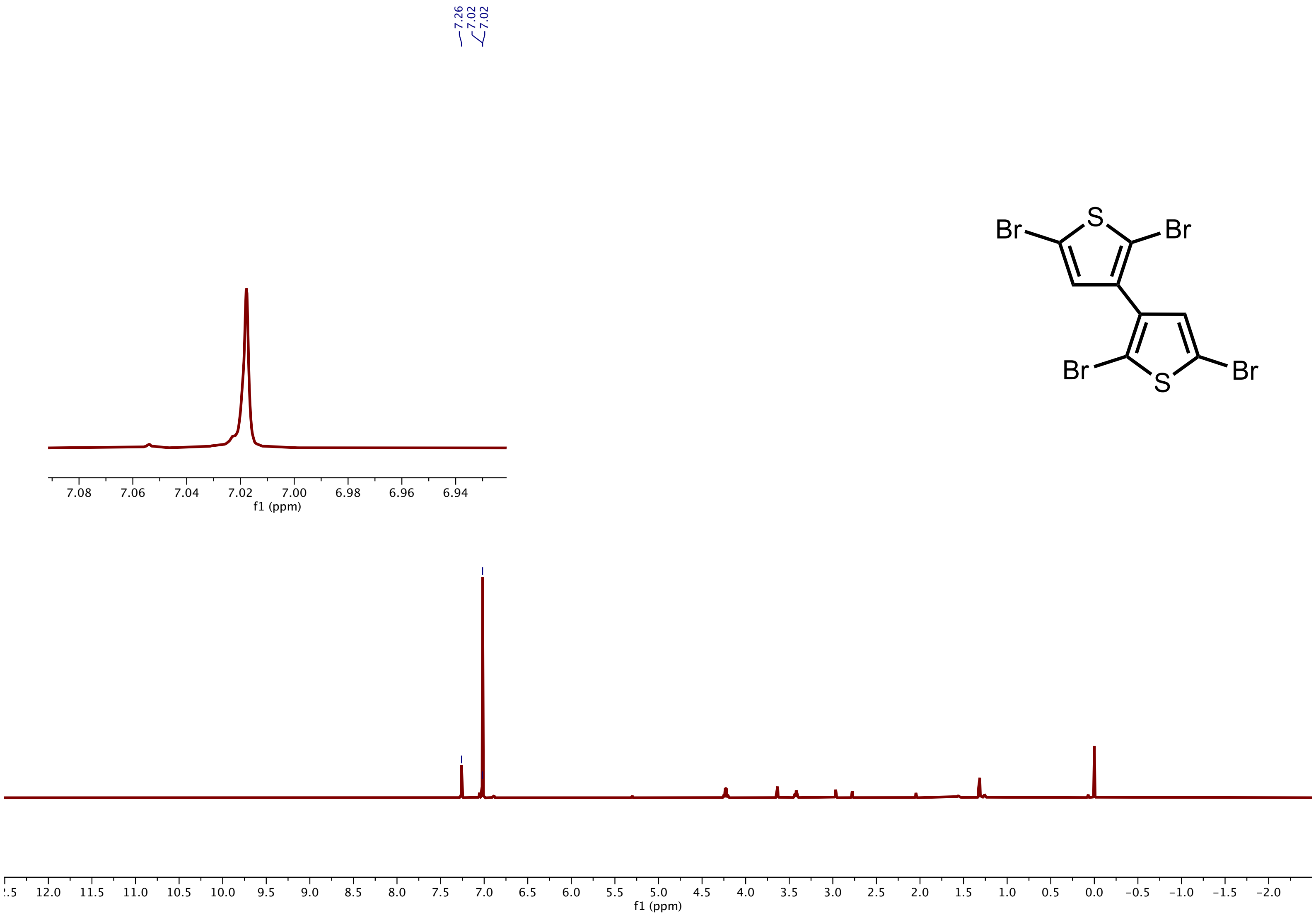

^{1}H NMR spectrum (500 MHz, $CDCl_3$, 25 °C) of 2,2',5,5'-tetrabromo-3,3'-bithiophene.

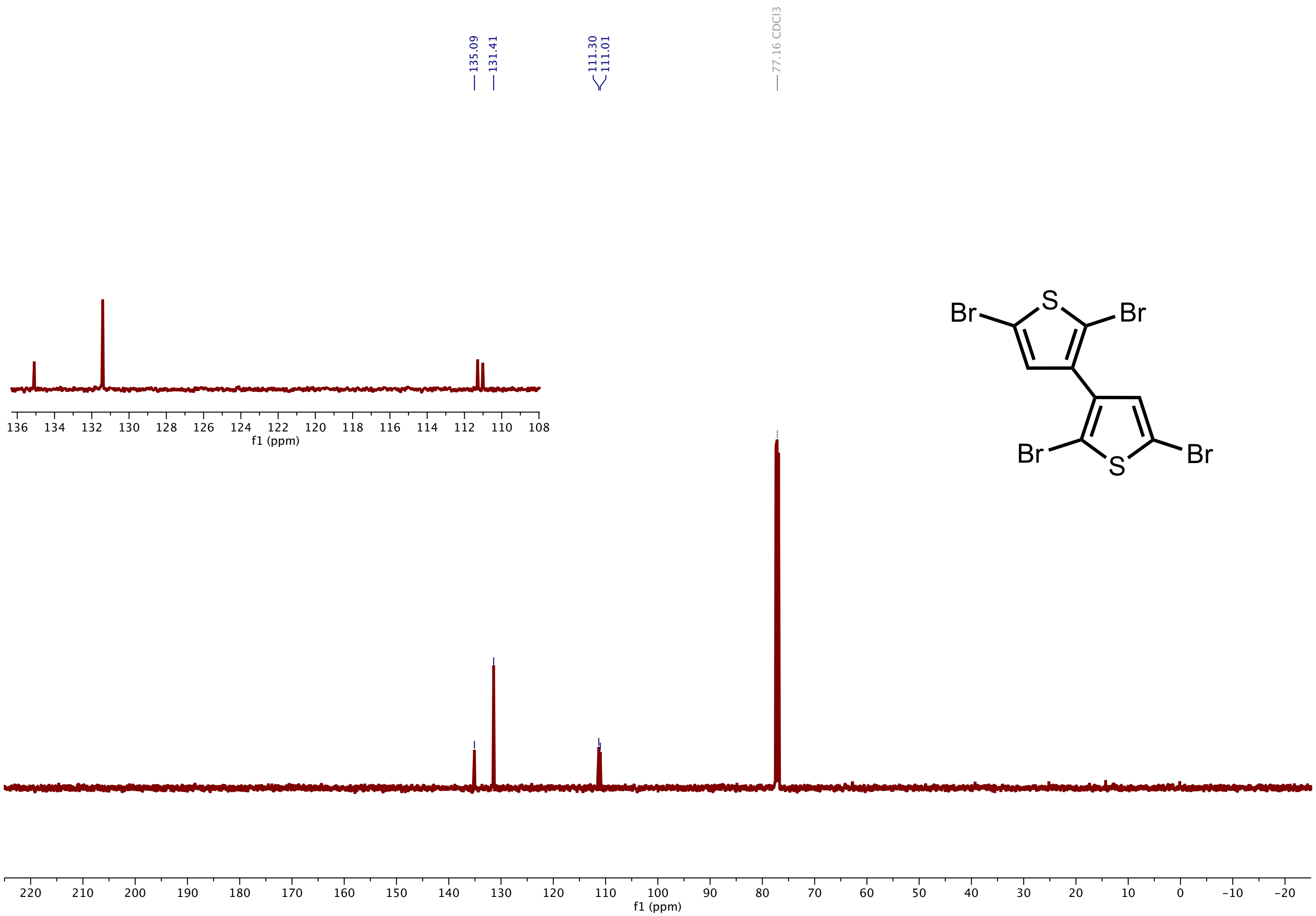

^{13}C NMR spectrum (126 MHz, $CDCl_3$, 25 °C) of 2,2',5,5'-tetrabromo-3,3'-bithiophene.

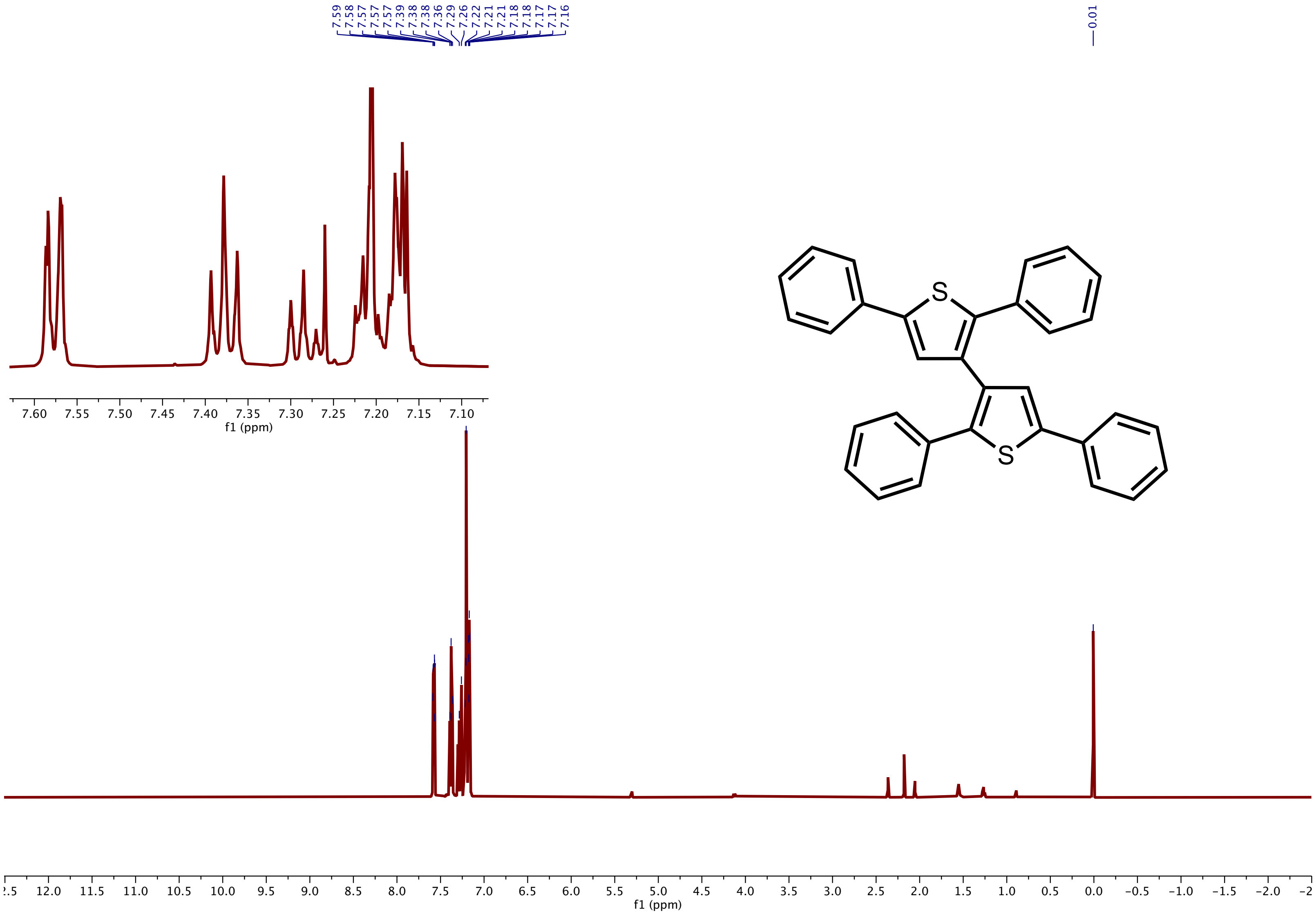

^{1}H NMR spectrum (500 MHz, $CDCl_3$, 25 °C) of **bis(PTh-H)**.

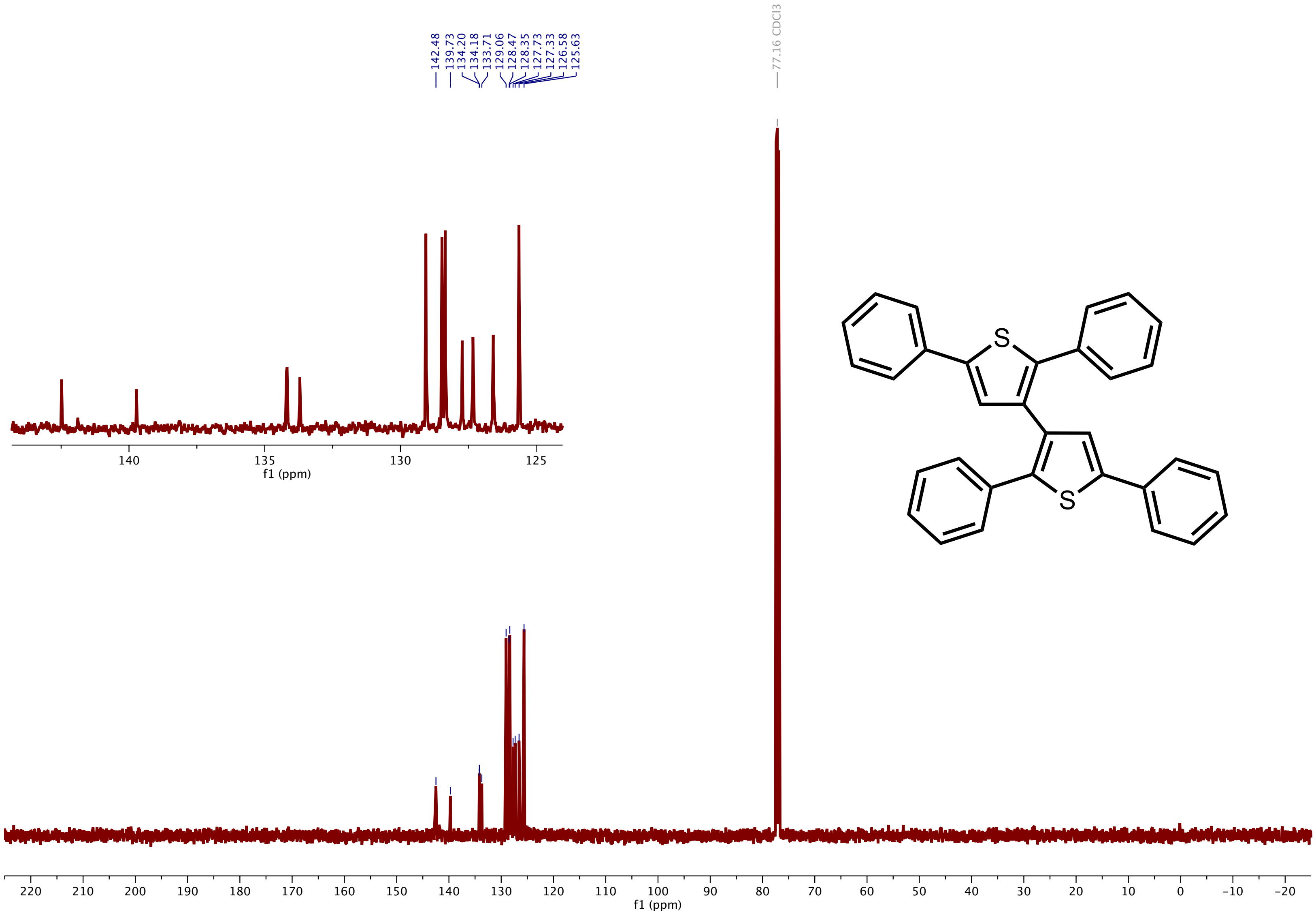

^{13}C NMR spectrum (126 MHz, $CDCl_3$, 25 °C) of **bis(PTh-H)**.

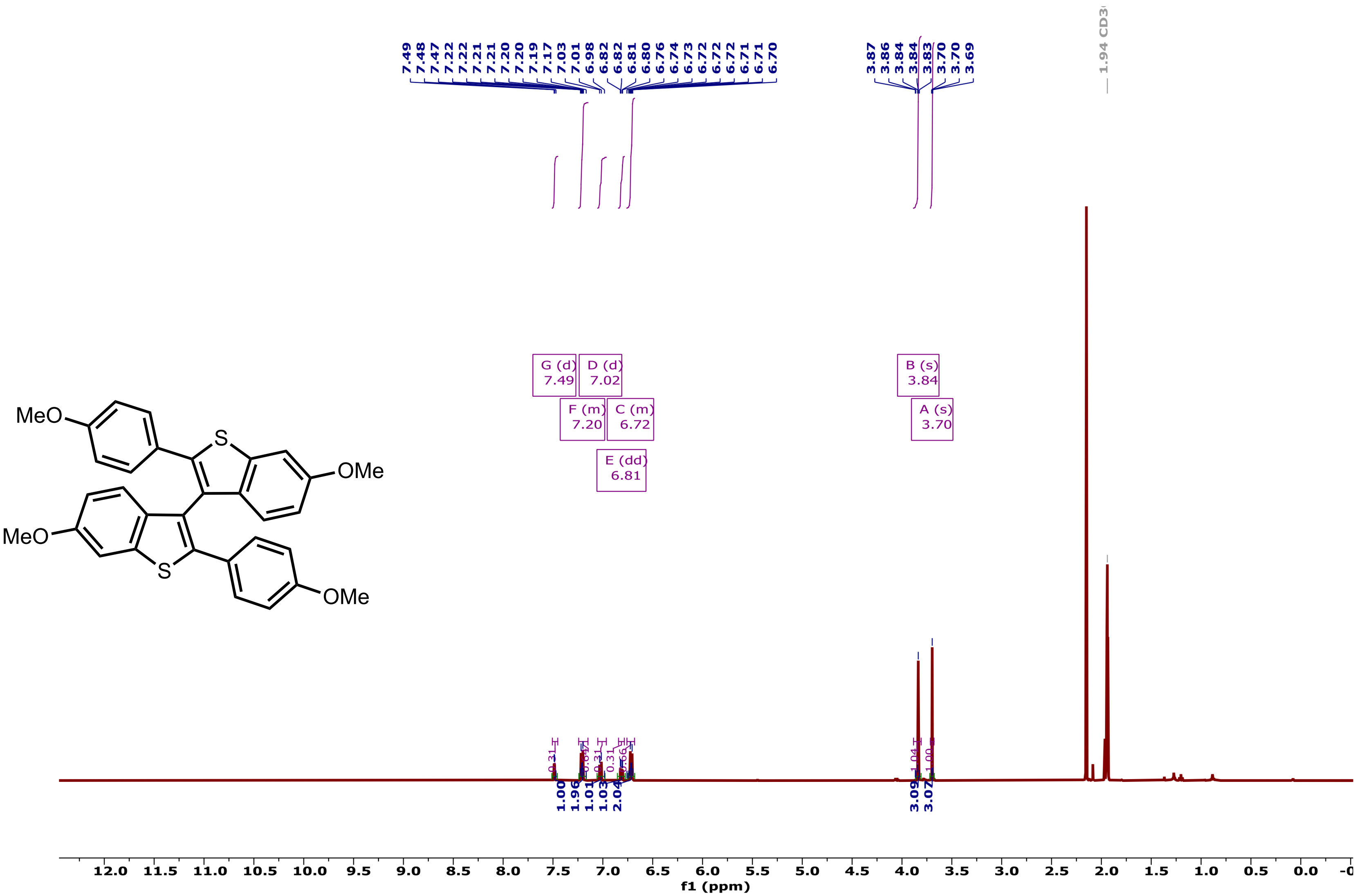

^{1}H NMR spectrum (500 MHz, CD_3CN, 25 °C) of **bis(BTh)**.

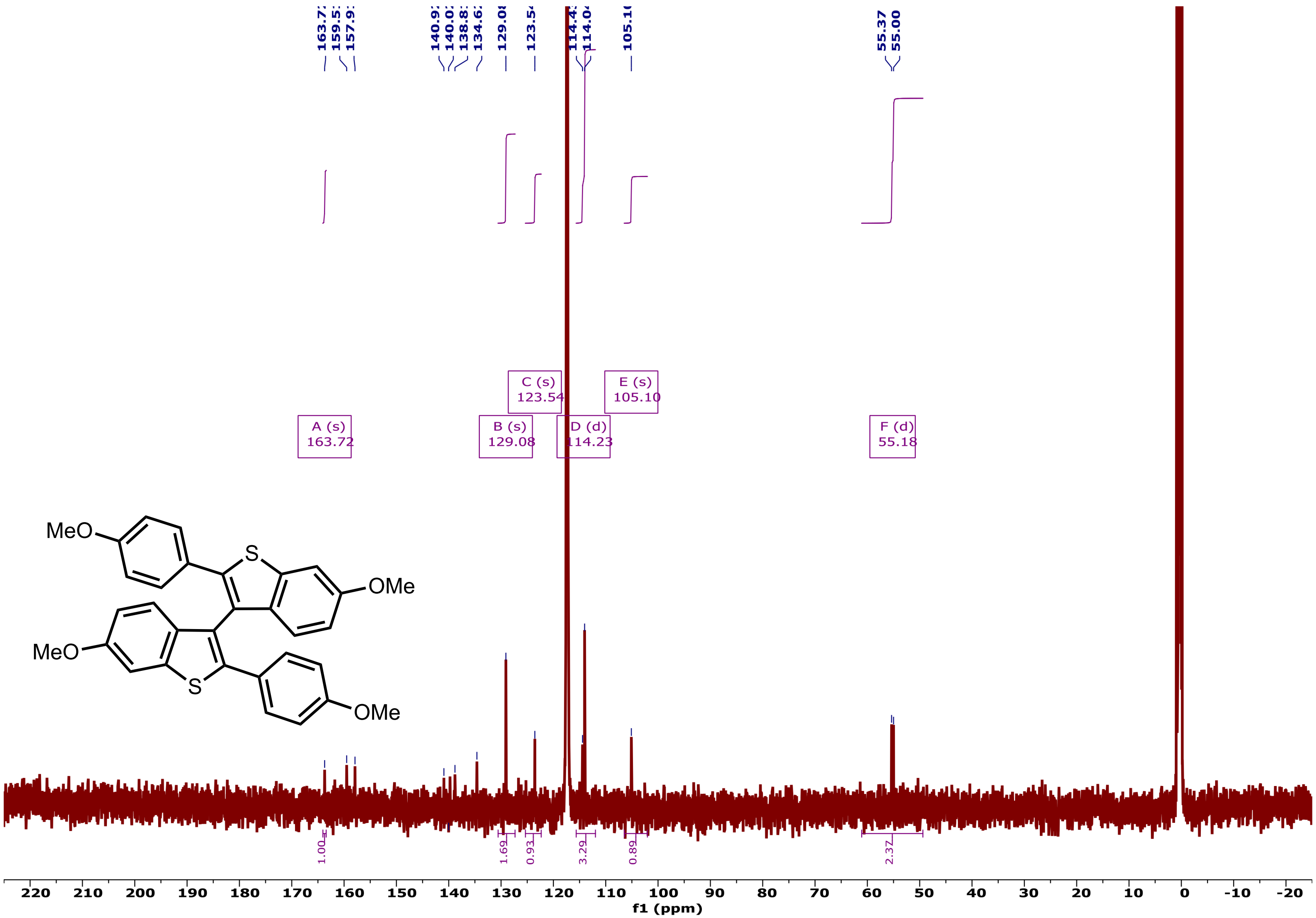

^{13}C NMR spectrum (399.78 MHz, CD_3CN, 25 °C) of **bis(BTh)**.

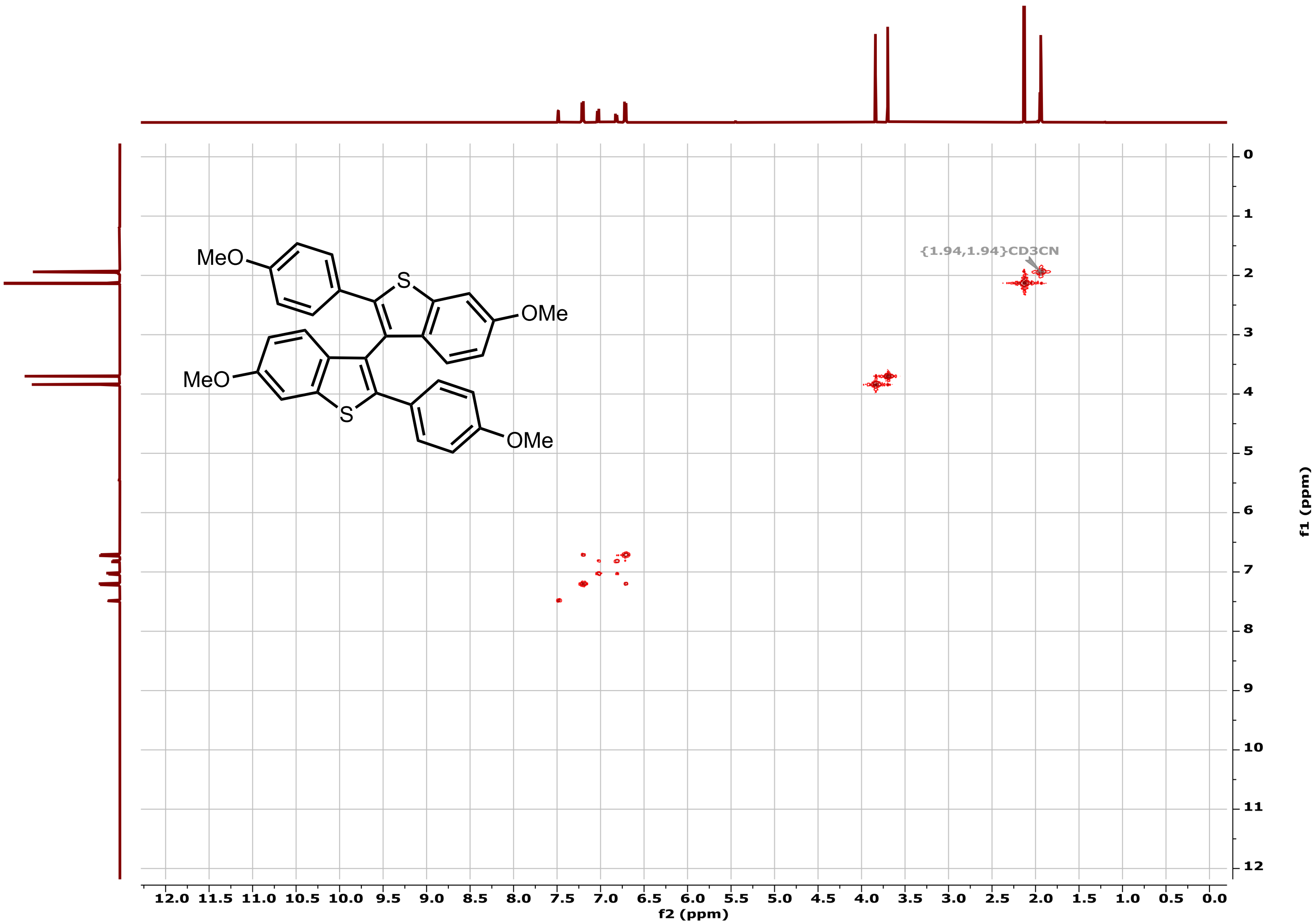

HMBC spectrum (^{1}H:500 MHz, ^{13}C: 126 MHz, CD_3CN, 25 °C) of **bis(BTh)**.

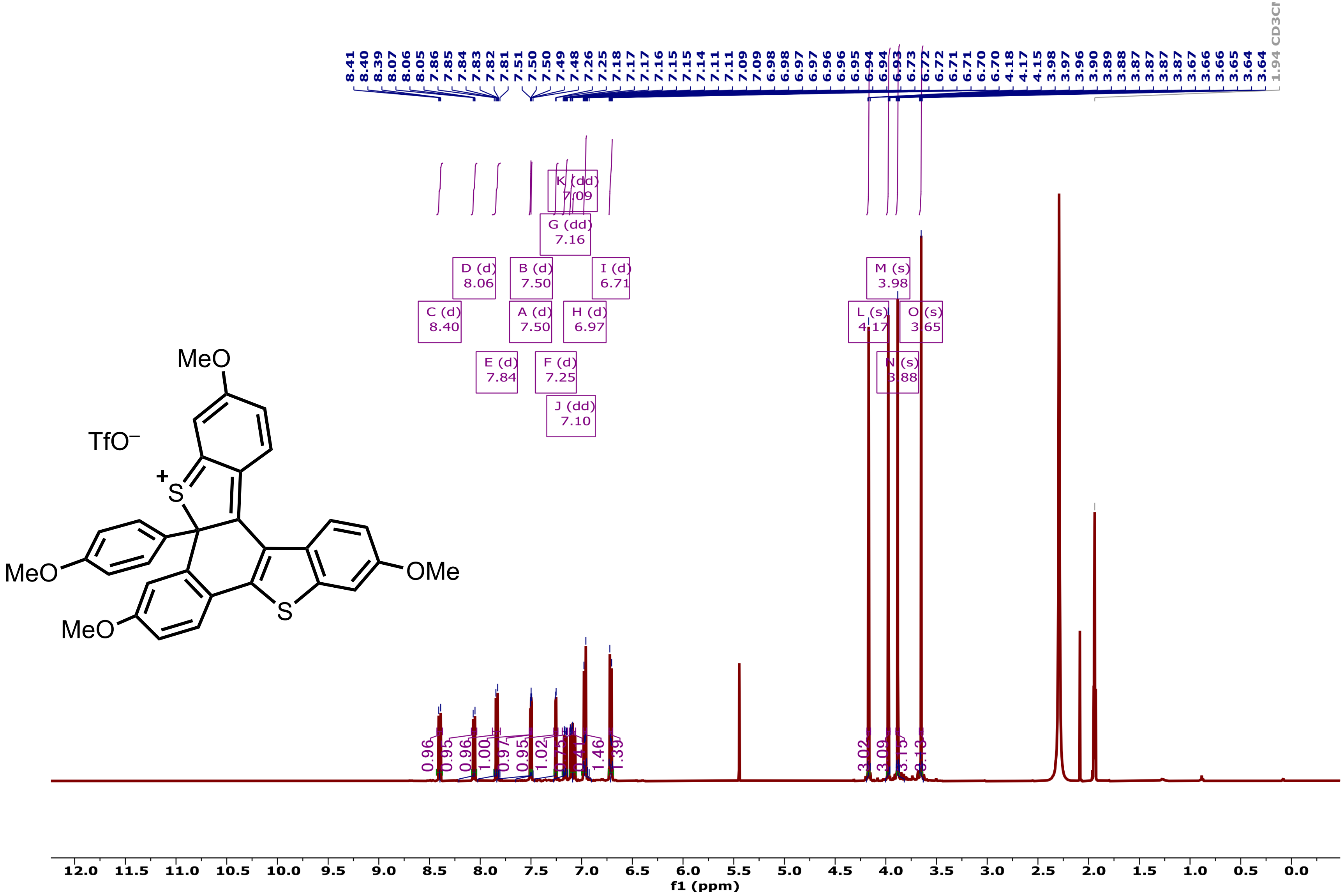

^{1}H NMR spectrum (500 MHz, CD_3CN, 25 °C) of **[BTh$_2$][TfO]**.

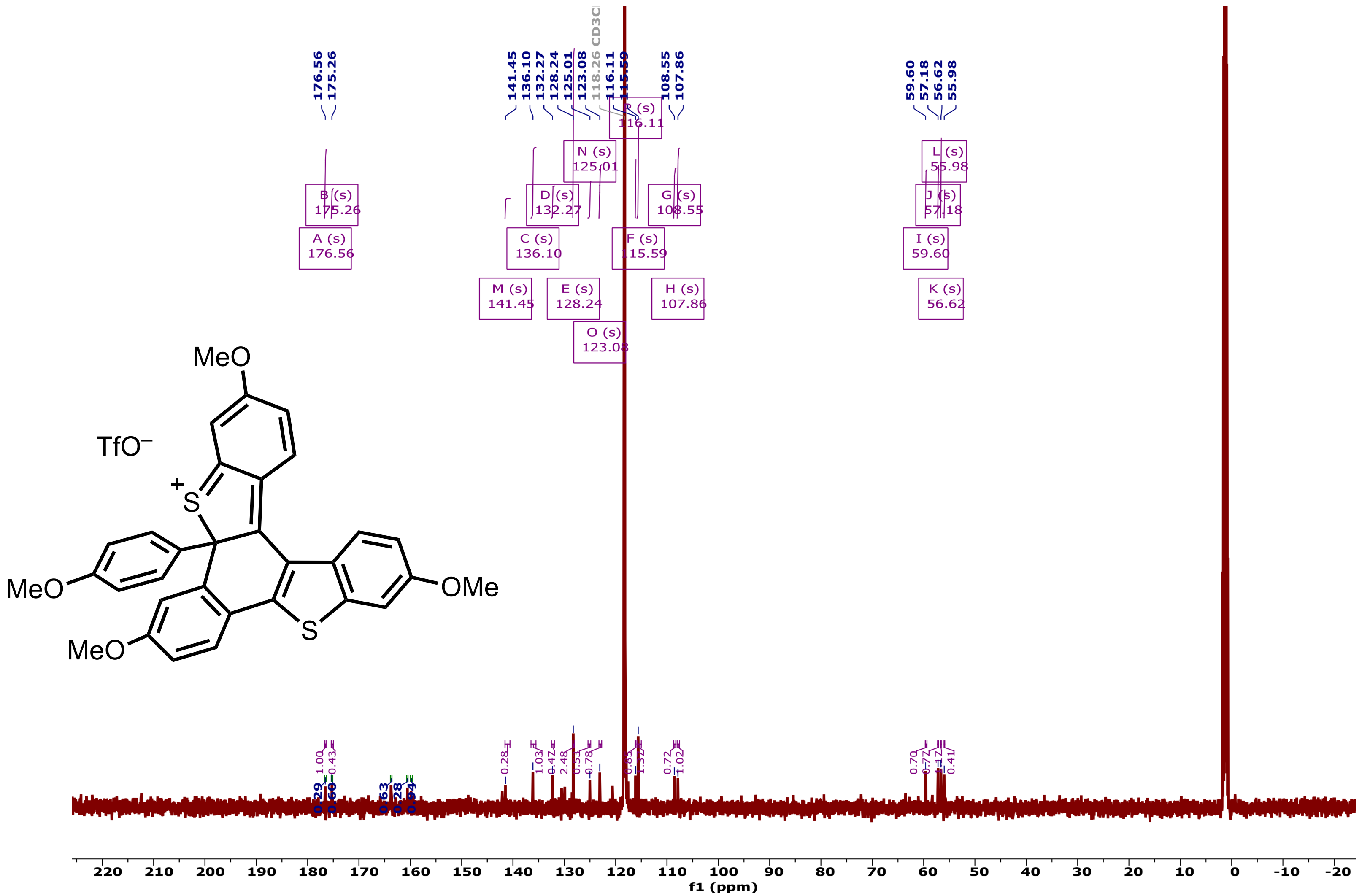

^{13}C NMR spectrum (126 MHz, CD_3CN, 25 °C) of **[BTh_2][TfO]**.

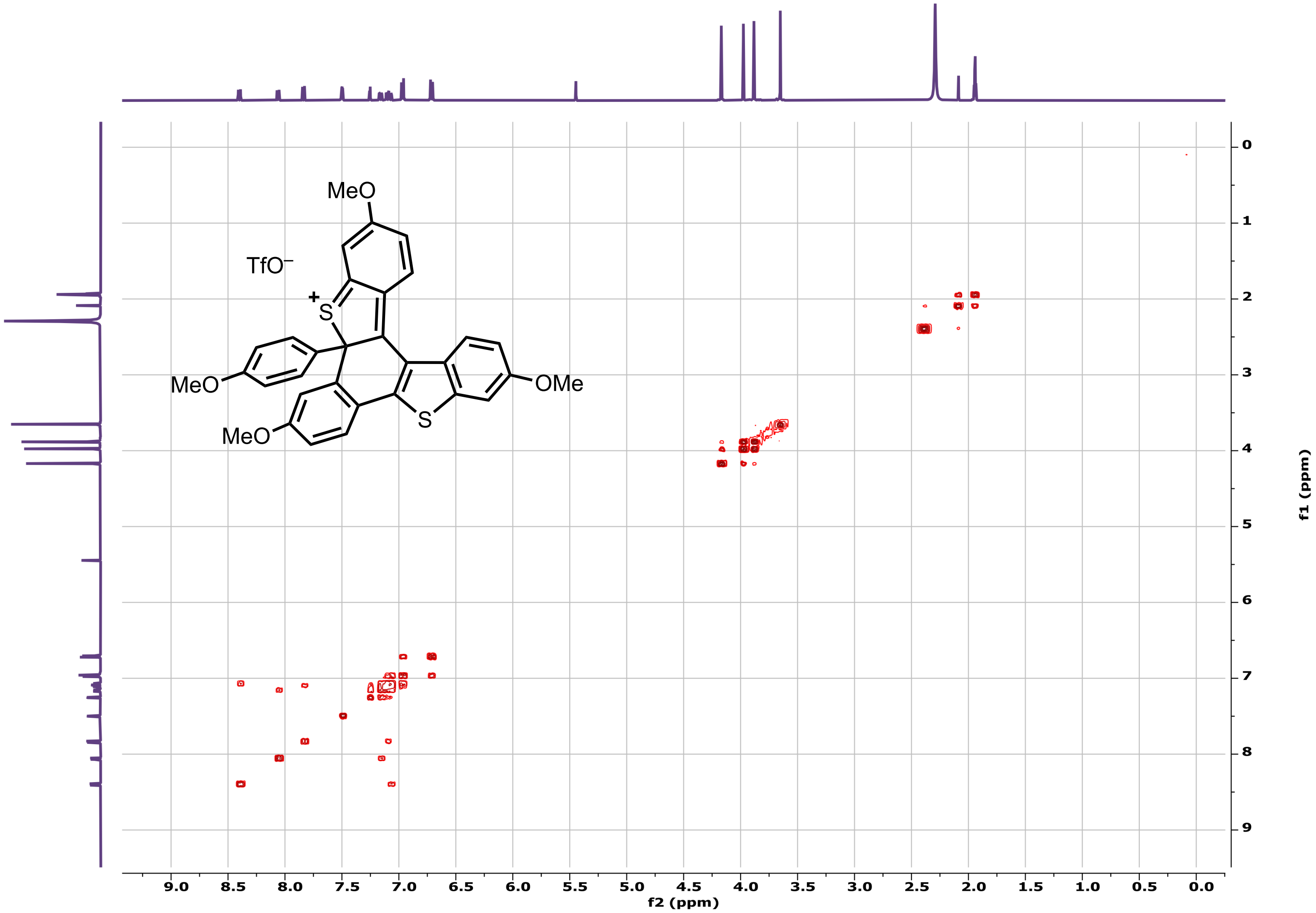

HMBC spectrum of of **[BTh$_2$][TfO]**.

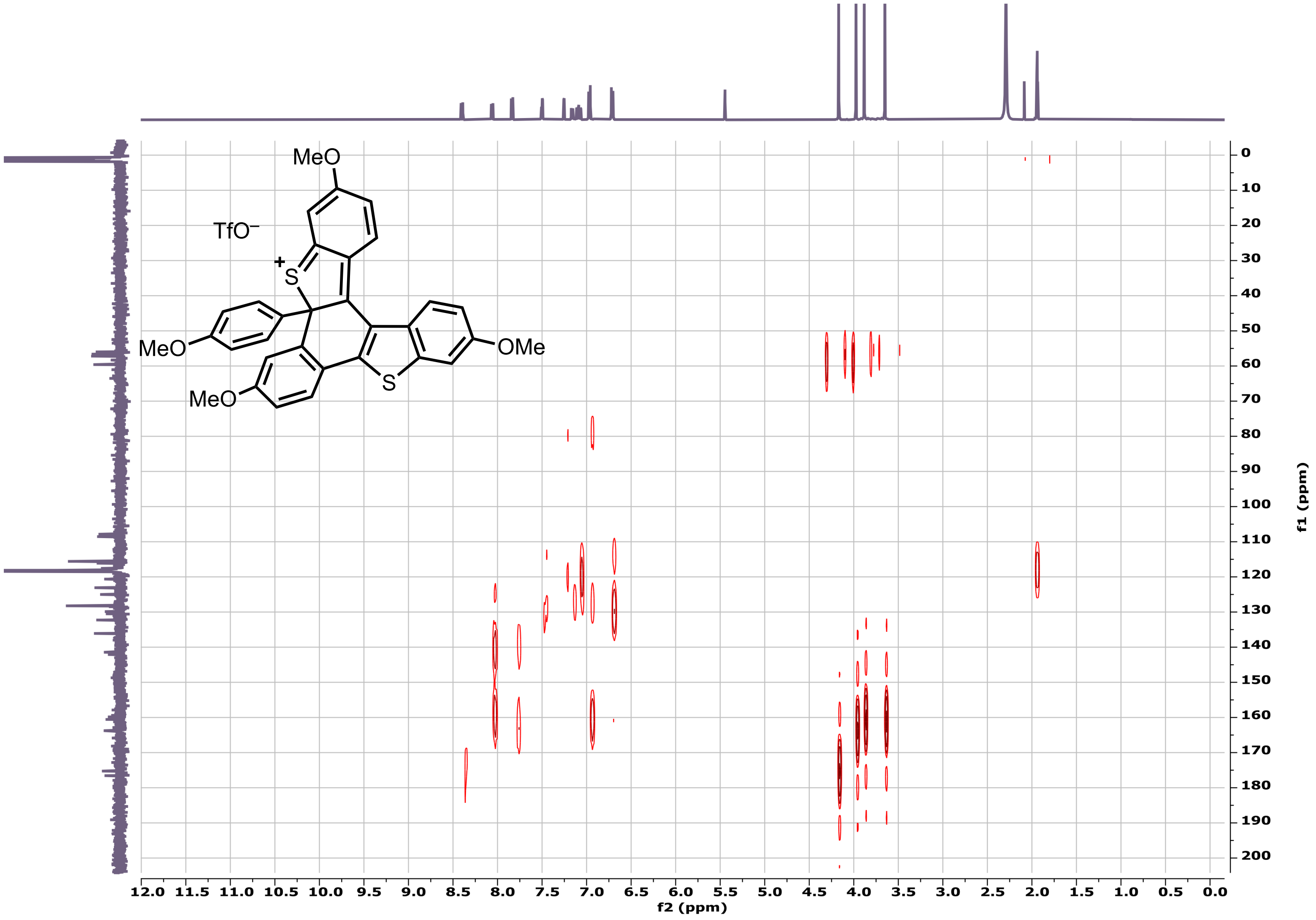

HMBC spectrum of **[BTh2][TfO]** enlarged at aromatic region of ^{1}H NMR.

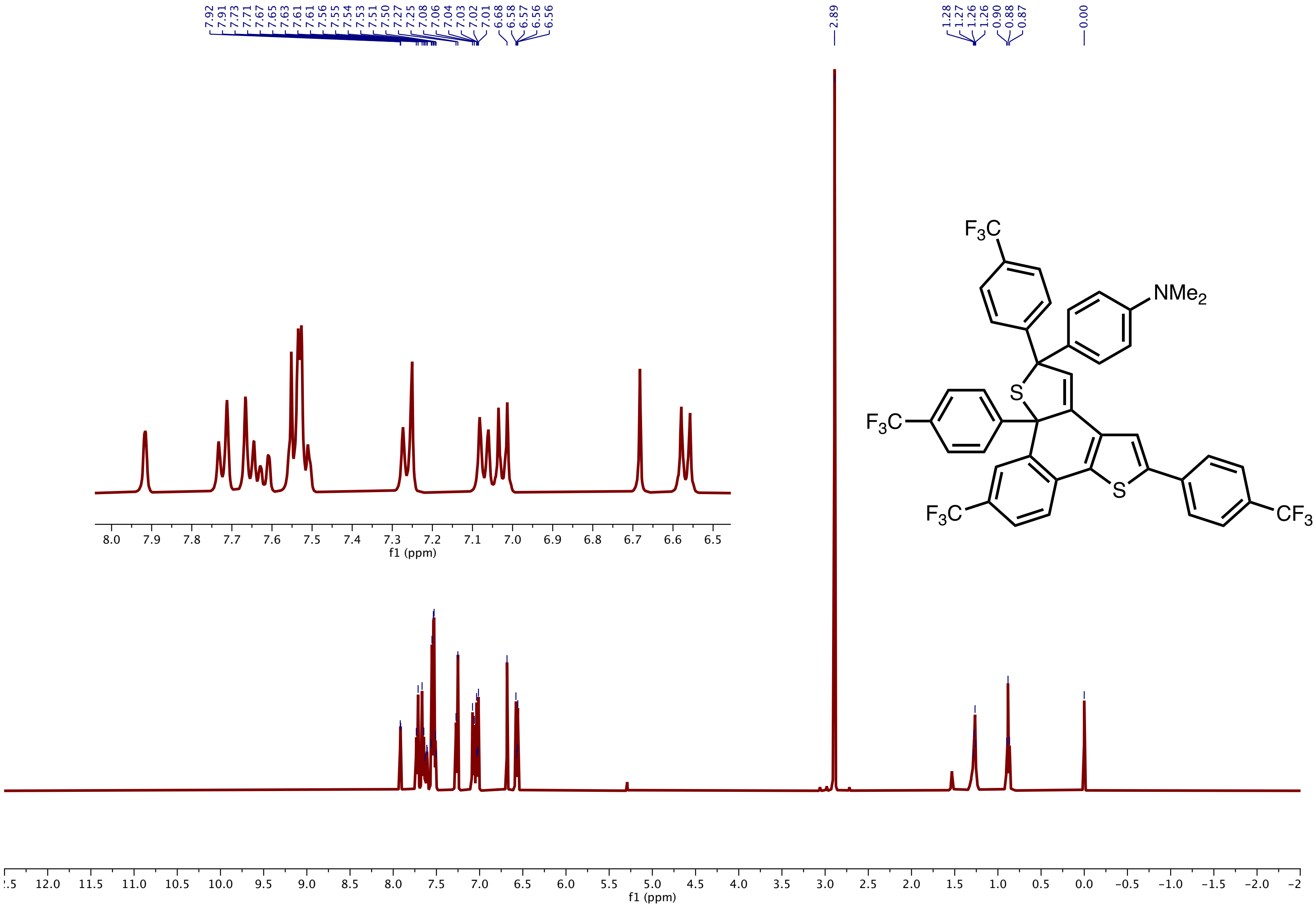

^1H NMR spectrum (399.78 MHz, $CDCl_3$, 25 °C) of **1**

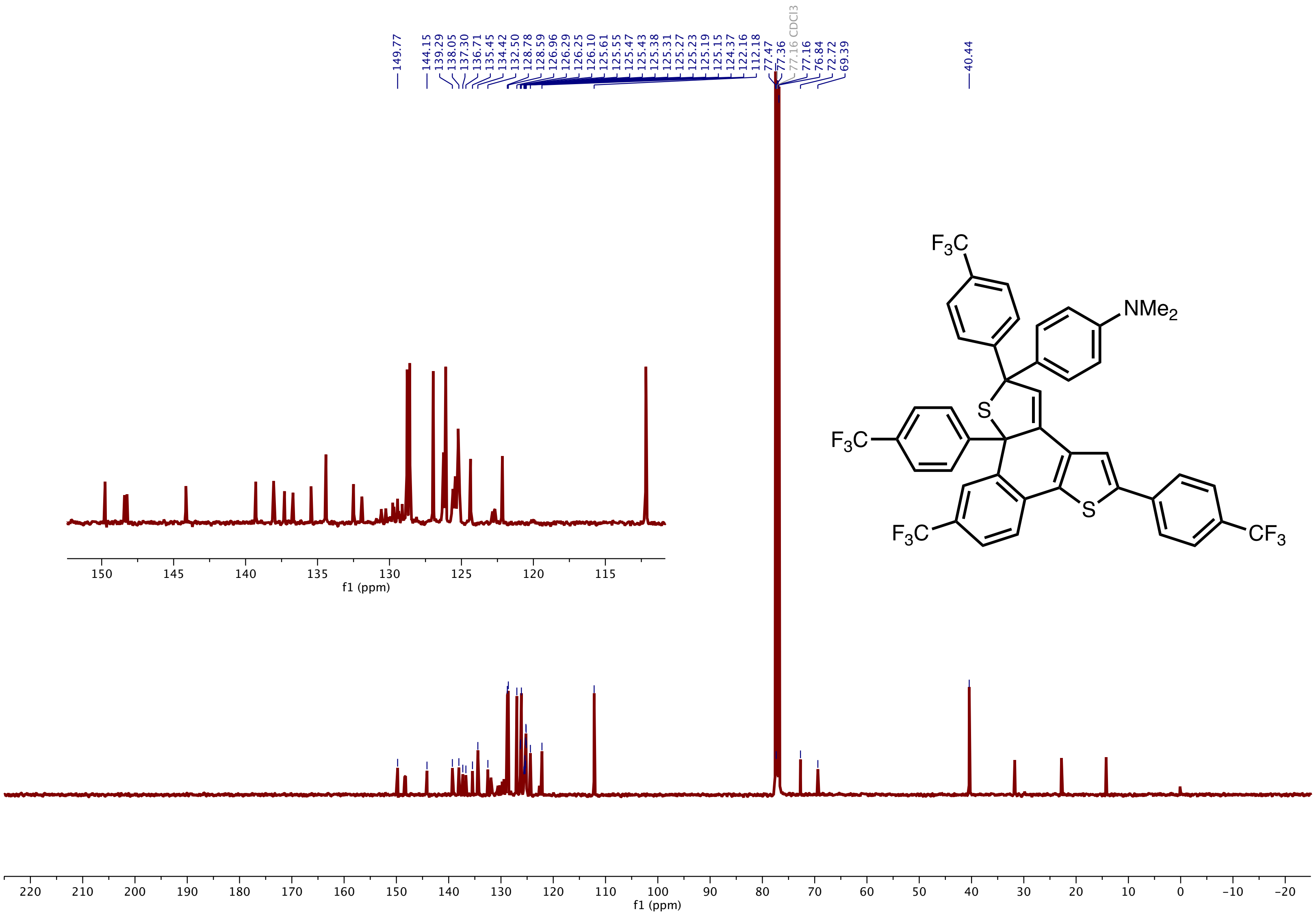

^{13}C NMR spectrum (100.53 MHz, $CDCl_3$, 25 °C) of **1**.

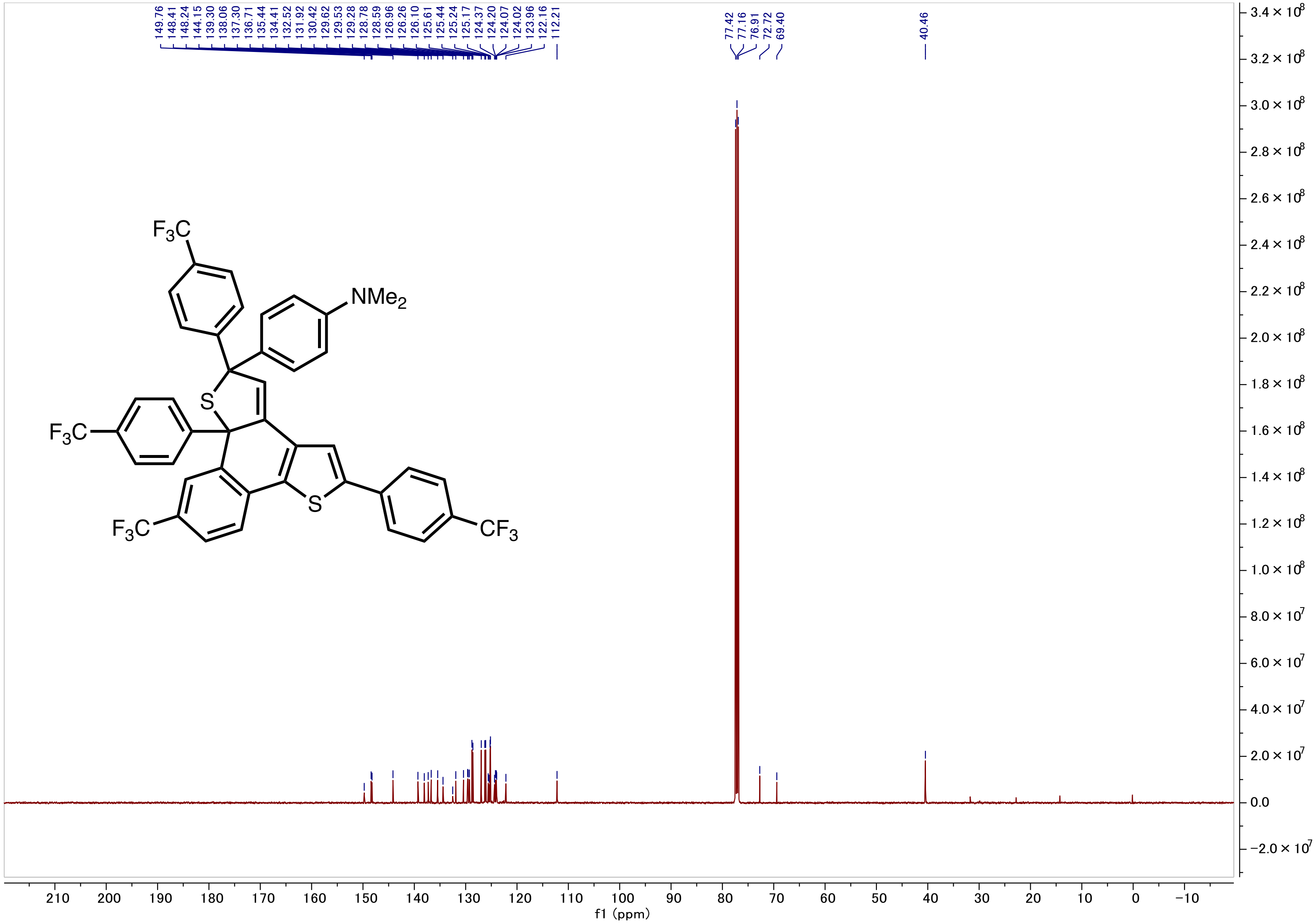

$^{13}C\{^{19}F\}$ NMR spectrum (126 MHz, $CDCl_3$, 25 °C) of **1**.

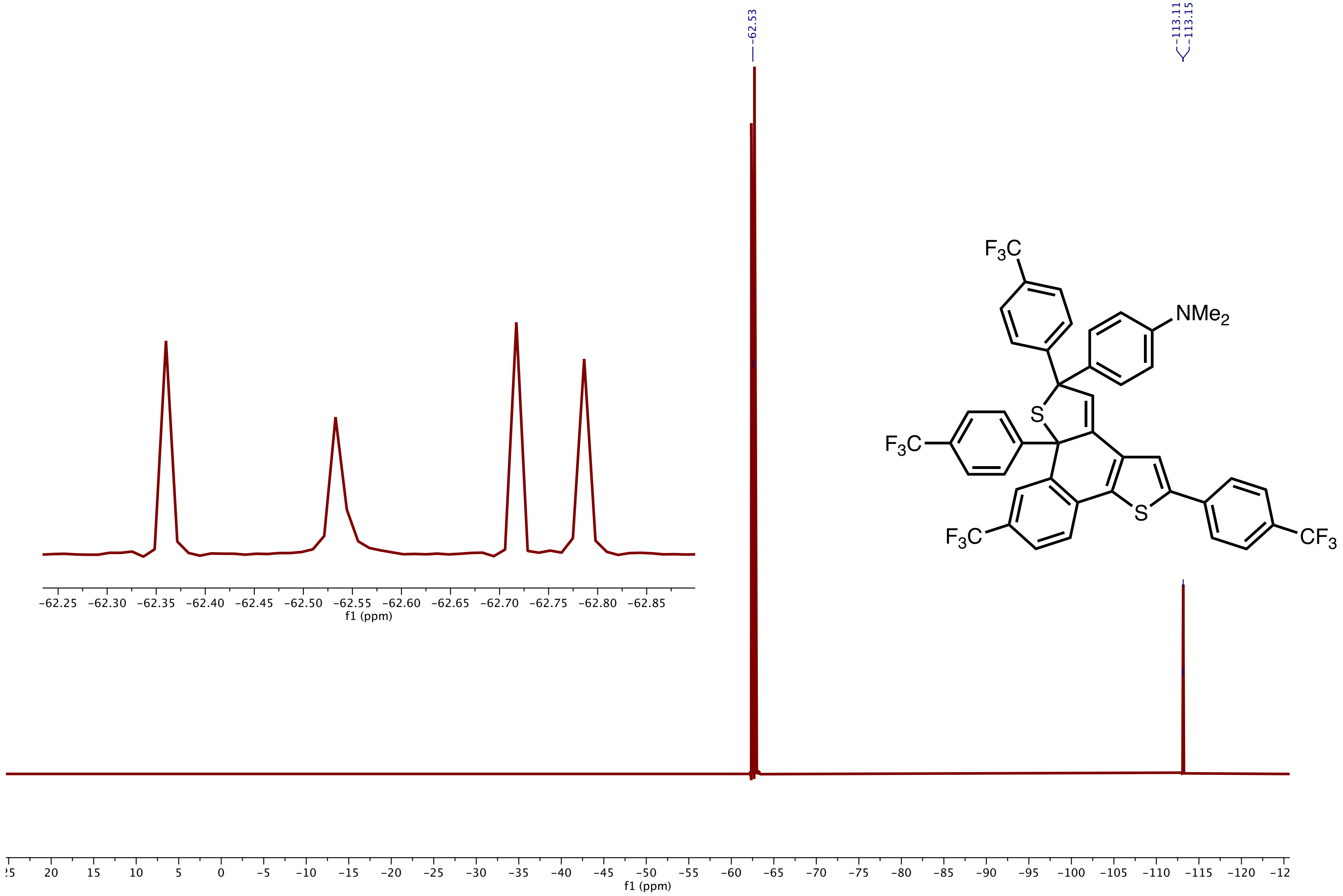

^{19}F NMR spectrum (376.46MHz, $CDCl_3$, 25 °C) of **1**.

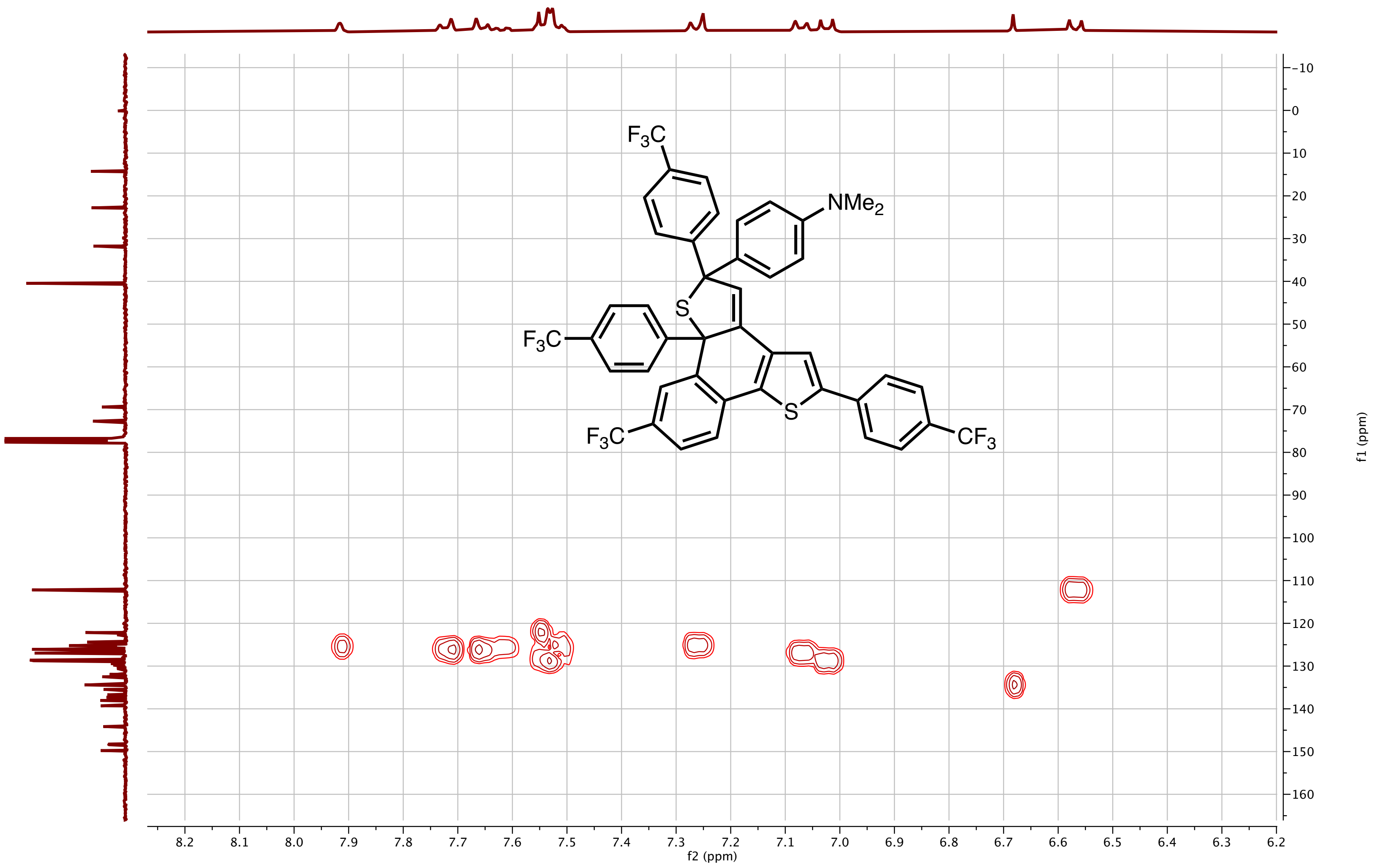

HMQC spectrum (^{1}H:399.78 MHz, ^{13}C: 100.53 MHz, $CDCl_3$, 25 °C) of **1** (enlarged at the aromatic region of ^{1}H NMR).

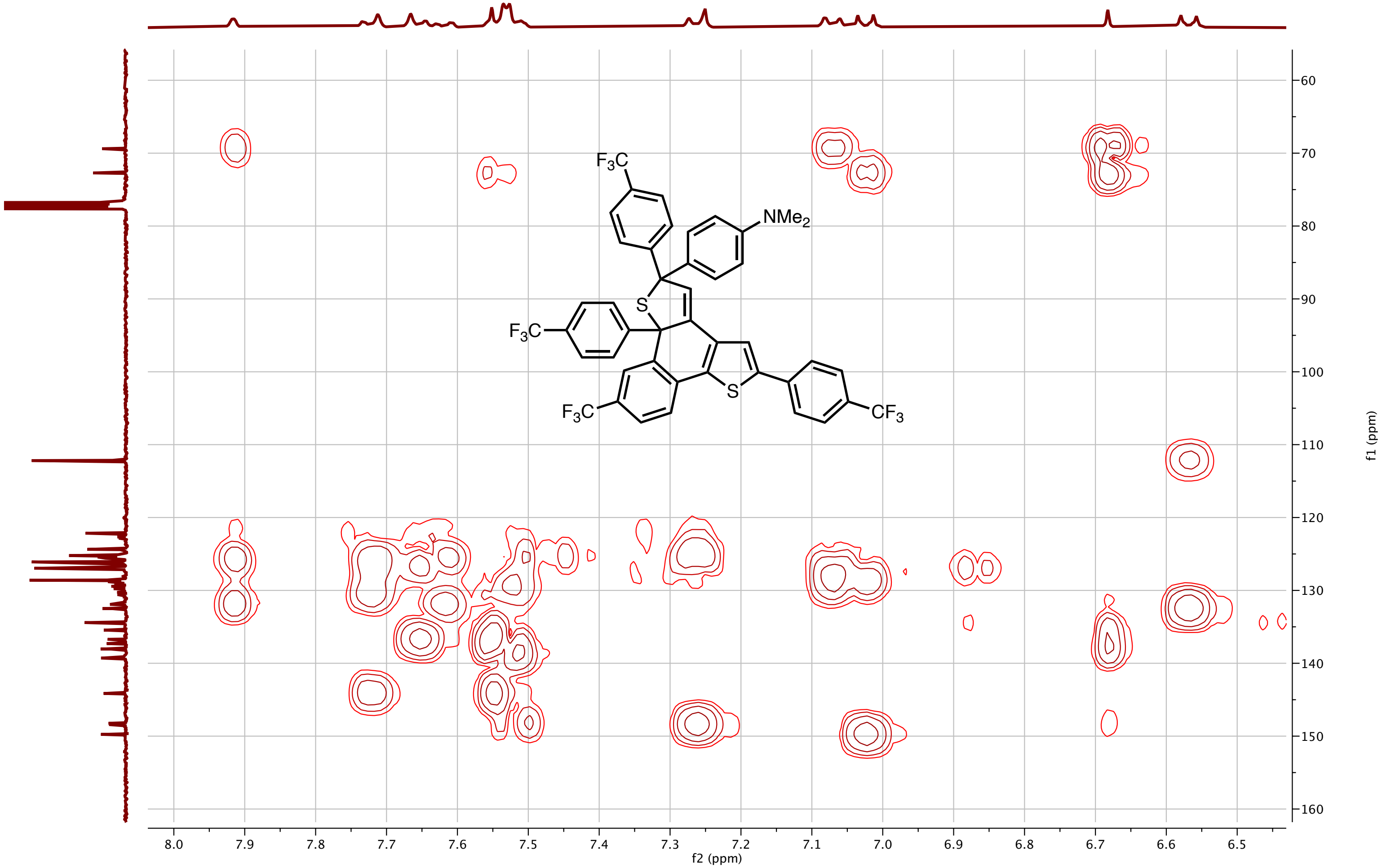

HMBC spectrum (^{1}H:399.78 MHz, ^{13}C: 100.53 MHz, $CDCl_3$, 25 °C) of **1** (enlarged at the aromatic region of ^{1}H NMR)
